# Loss of the nuclear Wnt pathway effector TCF7L2 promotes migration and invasion of human colorectal cancer cells

**DOI:** 10.1038/s41388-020-1259-7

**Published:** 2020-03-20

**Authors:** Janna Wenzel, Katja Rose, Elham Bavafaye Haghighi, Constanze Lamprecht, Gilles Rauen, Vivien Freihen, Rebecca Kesselring, Melanie Boerries, Andreas Hecht

**Affiliations:** 1 Institute of Molecular Medicine and Cell Research, Faculty of Medicine, University of Freiburg, Stefan-Meier-Str. 17, 79104 Freiburg, Germany; 2 Faculty of Biology, University of Freiburg, Schänzlestraße 1, 79104 Freiburg, Germany; 3 Institute of Medical Bioinformatics and Systems Medicine, Medical Center, Faculty of Medicine, University of Freiburg, Breisacherstr. 153, 79104 Freiburg, Germany; 4 Institute of Physics, University of Freiburg, Hermann-Herder-Str. 3a, 79104 Freiburg, Germany; 5 BIOSS Centre for Biological Signalling Studies, University of Freiburg, Schänzlestraße 18, 79104 Freiburg, Germany; 6 Freiburg Center for Interactive Materials and Bioinspired Technology (FIT), University of Freiburg, Georges-Köhler-Allee 105, 79110 Freiburg, Germany; 7 Department of General and Visceral Surgery, Center for Surgery, Medical Center, Faculty of Medicine, University of Freiburg, Hugstetter Straße 55, 79106 Freiburg, Germany; 8 German Cancer Consortium (DKTK), Hugstetter Straße 55, 79106 Freiburg, Germany; 9 German Cancer Research Center (DKFZ), Im Neuenheimer Feld 280, 69120 Heidelberg, Germany

**Keywords:** Mitosis, Cancer genomics, Growth factor signalling, Extracellular matrix

## Abstract

The transcription factor TCF7L2 is indispensable for intestinal tissue homeostasis where it transmits mitogenic Wnt/β-Catenin signals in stem and progenitor cells, from which intestinal tumors arise. Yet, *TCF7L2* belongs to the most frequently mutated genes in colorectal cancer (CRC), and tumor-suppressive functions of TCF7L2 were proposed. This apparent paradox warrants to clarify the role of *TCF7L2* in colorectal carcinogenesis. Here, we investigated *TCF7L2* dependence/independence of CRC cells and the cellular and molecular consequences of *TCF7L2* loss-of-function. By genome editing we achieved complete *TCF7L2* inactivation in several CRC cell lines without loss of viability, showing that CRC cells have widely lost the strict requirement for *TCF7L2*. *TCF7L2* deficiency impaired G1/S progression, reminiscent of the physiological role of TCF7L2. In addition, *TCF7L2*-negative cells exhibited morphological changes, enhanced migration, invasion, and collagen adhesion, albeit the severity of the phenotypic alterations manifested in a cell-line-specific fashion. To provide a molecular framework for the observed cellular changes, we performed global transcriptome profiling and identified gene-regulatory networks in which TCF7L2 positively regulates the proto-oncogene *MYC*, while repressing the cell cycle inhibitors *CDKN2C/CDKN2D*. Consistent with its function in curbing cell motility and invasion, *TCF7L2* directly suppresses the pro-metastatic transcription factor *RUNX2* and impinges on the expression of cell adhesion molecules. Altogether, we conclude that the proliferation-stimulating activity of TCF7L2 persists in CRC cells. In addition, TCF7L2 acts as invasion suppressor. Despite its negative impact on cell cycle progression, *TCF7L2* loss-of-function may thereby increase malignancy, which could explain why *TCF7L2* is mutated in a sizeable fraction of colorectal tumors.

## Introduction

Aberrant Wnt/β-Catenin pathway activity plays a crucial role in virtually every aspect of colorectal carcinogenesis [1], and more than 90% of all colorectal tumors carry mutations in either tumor-suppressive or oncogenic Wnt/β-Catenin pathway components [2]. These tumor-promoting lesions interfere with the regulated activity of the Wnt/β-Catenin pathway and thereby affect proliferation, migration, invasion, and tumor initiation capacity of colorectal cancer (CRC) cells [1]. A mainstay of Wnt/β-Catenin signaling are changes in gene expression. These are predominantly elicited through the interaction of β-Catenin with members of the T-cell factor/lymphoid enhancer binding factor (TCF/LEF) family [3]. In mammals, this family consists of LEF1, TCF7, TCF7L1, and TCF7L2. All TCF/LEF family members possess as common structural features interaction domains for β-Catenin and Transducin-like Enhancer of Split/Groucho-related genes (TLE/GRG) corepressors, and an HMG-box DNA-binding motif [3]. Structural diversity outside these domains equips TCF/LEF family members with individual gene-regulatory capacity, and allows them to carry out both redundant and nonredundant functions as nuclear effectors of the Wnt/β-Catenin pathway [3–5].

Wnt/β-Catenin signaling governs multiple aspects of cellular dynamics and tissue architecture in the developing gastrointestinal tract and in adult intestinal tissue homeostasis [1, 6]. Particularly relevant for colorectal carcinogenesis are the stimulation of cell proliferation and the maintenance of intestinal stem cells [6] from which colorectal tumors appear to arise [7]. In the healthy gut, these Wnt/β-Catenin pathway functions are executed exclusively via TCF7L2 [8–11]. Accordingly, inactivation of the *Tcf7l2* gene in mouse models and intestinal organoids is lethal due to diminished mitogenic activity and depletion of stem and progenitor cells [8–11]. There is also evidence that *Tcf7l2* is indispensable for tumor initiation [11] which agrees well with the positive regulation of several oncogenes by TCF7L2 [12–16].

Its essential function in stimulating cell proliferation in the healthy murine intestine and its role in transmitting oncogenic Wnt/β-Catenin signals in mouse tumor models seemingly qualify TCF7L2 as a tumor-promoting factor also in human colorectal carcinogenesis. This view contrasts with the frequent occurrence of *TCF7L2* loss-of-function mutations in CRC genomes [2, 17, 18], arguing that TCF7L2 activity may rather be tumor-suppressive. Indeed, TCF7L2 was claimed to function as haploinsufficient tumor suppressor in mice [9], and to restrict human CRC cell cycle progression [9, 19]. However, both findings were recently challenged [11]. Thus, the role of *TCF7L2* in human CRC remains ambiguous. Specifically, it is unknown to which extent CRC cells tolerate complete absence of *TCF7L2*, and how this would affect cellular phenotypes. Likewise, a sustainable explanation for the high *TCF7L2* mutation frequency is lacking. To address these issues, we systematically knocked-out *TCF7L2* in CRC cell lines. Our results show that the vital necessity for *TCF7L2* in healthy intestinal cells is broadly lost in the course of colorectal carcinogenesis. Even though TCF7L2-negative cells exhibit delayed G1/S transition, they are more migratory and invasive, and show enhanced collagen adhesion. Concomitantly, TCF7L2 deficiency disturbs gene-regulatory networks comprising cell cycle regulators, the pro-metastatic transcription factor *RUNX2*, and multiple cell adhesion molecules. Apparently, *TCF7L2* has properties of a migration/invasion suppressor, which provides a biological rationale for the frequent mutation of *TCF7L2* in CRC genomes.

## Results

### Human CRC cells survive without TCF7L2

**Fig. 1:**
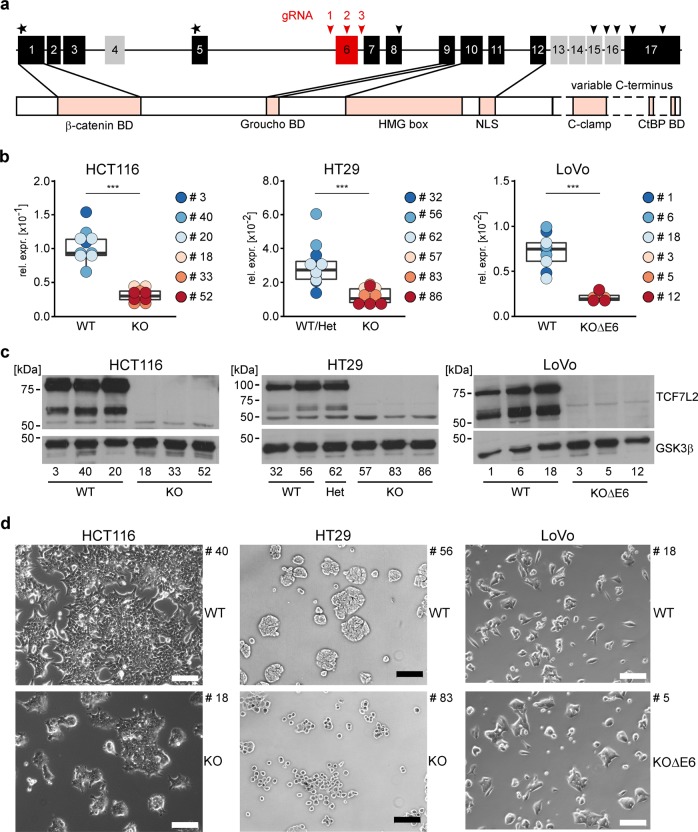
CRC cell lines are viable without TCF7L2. **a** Top: scheme of the *TCF7L2* gene with its 17 exons (numbered boxes). Constitutively expressed exons are colored in black and red, alternatively spliced exons in gray. Asterisks mark start codons. Black arrowheads denote stop codons whose usage depends on the exon composition of alternatively spliced transcripts. The locations of gRNAs are indicated by red arrowheads. For HCT116 and HT29 cells, the gRNA g2 was used to disrupt the constitutive exon 6 (red box). For LoVo cells, gRNAs g1 and g3 were used to delete exon 6. Bottom: TCF7L2 protein structure. Functionally important domains are marked and connected to the exon(s) by which they are encoded. HMG-box DNA-binding domain. NLS nuclear localization sequence. BD binding domain. **b** The mRNA expression of *TCF7L2* was analyzed by qRT-PCR in cell clones with biallelic wildtype (WT), heterozygous (Het), and biallelic knockout (KO) *TCF7L2* genes with intra-exon 6 mutations or exon 6 deletion (KOΔE6). *TCF7L2* transcript levels were normalized to *GAPDH*, and are displayed as relative expression (rel. expr.). Each dot represents an individual measurement, color of the dots identifies different cell clones. The box plots summarize and compare expression data from *TCF7L2*^WT/Het^, *TCF7L2*^KO^, and *TCF7L2*^KOΔE6^ cells. Linear mixed model (LMM) analysis was performed to assess significance (*n* = 3). **c** Nuclear extracts were generated from *TCF7L2*^WT/Het^, *TCF7L2*^*KO*^, and *TCF7L2*^KOΔE6^ clones and western blot analysis was performed to detect TCF7L2 expression. GSK3β was used as loading control. Molecular weights are given in kDa. Representative results from one of three independent biological replicates are shown. **d** Representative micrographs from one of three independent biological replicates showing the indicated *TCF7L2*^WT^, *TCF7L2*^KO^, and *TCF7L2*^KOΔE6^ cell clones 72 h after seeding the same starting numbers of cells. The scale bars represent 200 µm.

We confirmed that murine intestinal organoids do not survive inactivation of *Tcf7l2* (Supplementary Fig. S1). To test whether the essential function of *TCF7L2* is preserved in human CRC cells, we utilized the CRISPR/Cas9 system to target *TCF7L2* exon 6 (Fig. 1a) which is common to all known *TCF7L2* RNA isoforms [20]. Expression patterns of TCF/LEF family members in colorectal tumors deviate from the healthy intestinal epithelium and are highly variable, as evident from CRC transcriptome data (Supplementary Fig. S2a, b), and immunohistochemical stainings of case-matched normal and CRC tissue specimens (Supplementary Figs. S3, S4). Consistent with this, we observed that CRC cell lines express diverse combinations of TCF/LEF factors (Supplementary Fig. S2c, d). To take into account the variability of TCF/LEF expression, we therefore selected the three CRC cell lines HT29, HCT116, and LoVo for genome editing. Among these, HT29 cells express TCF7 and TCF7L2 (Supplementary Fig. S2c, d), reflecting native TCF/LEF expression in the normal mouse and human colonic epithelium (Supplementary Figs. S3–S5). HCT116 cells additionally express TCF7L1 (Supplementary Fig. S2c, d). LoVo cells express all four TCF/LEF family members (Supplementary Fig. S2c, d). Furthermore, the cell lines chosen cover a range of different CRC-associated lesions in the Wnt/β-Catenin, MAP kinase, TP53, and TGFβ pathways (Supplementary Table S1) [2, 21–23]. Irrespective of their TCF/LEF status and the respective mutations in CRC driver genes, we obtained multiple clones with biallelic inactivation of *TCF7L2* for all three cell lines (Fig. 1b, c; Supplementary Table S2). *TCF7L2* knockout (KO) clones showed strongly reduced *TCF7L2* transcription and complete absence of all TCF7L2 protein isoforms regardless of whether *TCF7L2* was inactivated by intra-exon 6 mutations (HCT116 and HT29 *TCF7L2*^KO^ cells) or exon 6 deletion (LoVo *TCF7L2*^KOΔE6^ cells). In contrast, a heterozygous (Het) HT29 cell clone carrying one *TCF7L2* wildtype (WT) and one KO allele, expressed TCF7L2 at levels indistinguishable from *TCF7L2*^WT^ cells. Notably, there was no compensatory upregulation of the other TCF/LEF family members in the absence of TCF7L2 (Supplementary Fig. S6). *TCF7L2* deficiency did not affect the growth pattern and morphology of LoVo *TCF7L2*^KOΔE6^ cells (Fig. 1d), but HCT116 *TCF7L2*^KO^ clones showed subtle morphological changes by forming more protrusions. HT29 *TCF7L2*^KO^ cultures exhibited more pronounced alterations and grew as isolated, round cells instead of generating dense cell clusters like HT29 *TCF7L2*^WT/Het^ clones. Furthermore, it appeared that HCT116 and HT29 *TCF7L2*^KO^ cultures expanded slower than *TCF7L2*^WT/Het^ cells (Fig. 1d). To ascertain that the phenotypic alterations observed with HCT116 and HT29 *TCF7L2*^KO^ cells were not due to off target effects or differences in KO strategies, we created additional *TCF7L2*-deficient HCT116 and HT29 cell clones by inflicting the same exon 6 deletion as in LoVo cells. This yielded HCT116 and HT29 *TCF7L2*^KOΔE6^ cell clones exhibiting morphological changes highly similar, if not identical, to *TCF7L2*^KO^ cells (Supplementary Fig. S7). As observed with the latter, no increase in expression of the other TCF/LEF family members was seen in HCT116 and HT29 *TCF7L2*^KOΔE6^ cell clones (Supplementary Fig. S8). Overall, we conclude that *TCF7L2* is not essential for the survival of at least some human CRC cells. Nonetheless, *TCF7L2* deficiency is accompanied by phenotypic changes in HCT116 and HT29 cells that manifest despite the presence of other TCF/LEF family members.

### Comprehensive transcriptome analyses of *TCF7L2*^KO^ clones reveal changes in Wnt signaling, proliferation, and adhesion

**Fig. 2:**
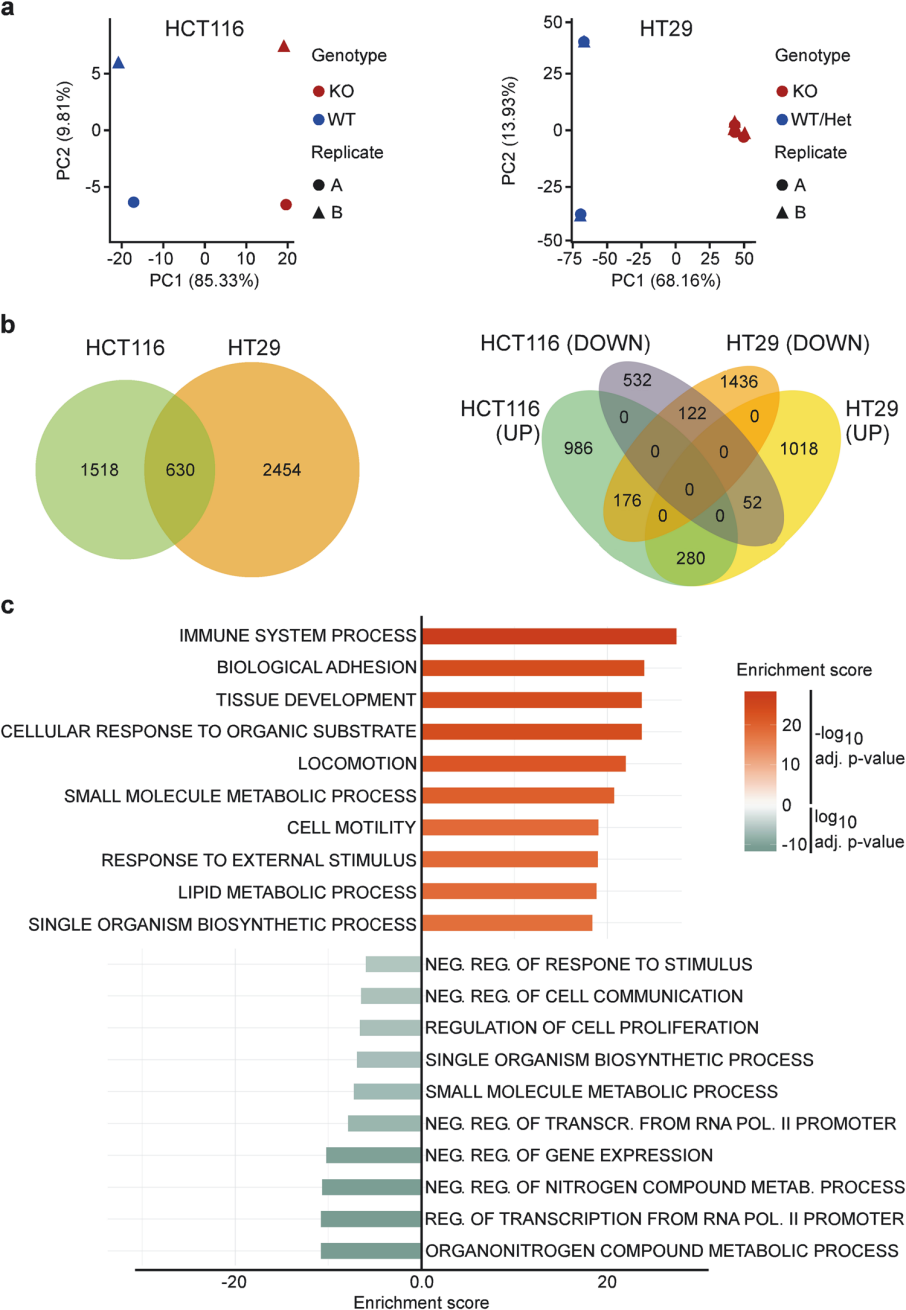
Comprehensive transcriptome analyses of *TCF7L2*^KO^ cells reveal commonly deregulated genes in HCT116 and HT29 cells. **a** The number of raw counts was determined after alignment of RNA-seq data. This process was conducted with HCT116 and HT29 cell clones with biallelic WT, KO, and Het *TCF7L2* genes (HCT116: WT #3, KO #18; HT29: WT #56, Het #62, KO #57, #83, #86) for each of two independent biological replicates. By applying principal component analysis (PCA) on the regularized-logarithm transformed values of the raw counts [69], the cell lines were depicted in two-dimensional PCA plots. The shape of the symbols identifies the replicates and their color indicates the *TCF7L2* genotype. **b** Comparison of *TCF7L2*-dependent gene expression in HCT116 and HT29 cells. Numbers of DEGs in *TCF7L2*^KO^ versus *TCF7L2*^WT/Het^ cells, and their overlap between HCT116 and HT29 cells are shown in the Venn diagram on the left. The Venn diagram on the right differentiates between cell-line-specific and HCT116/HT29-common up- and downregulated genes. **c** Functional enrichment analysis of gene sets was performed on the *TCF7L2*-dependent DEGs common to HCT116 and HT29 cells using Fisher’s exact test. The top ten positively and negatively enriched Gene Ontology (GO) terms for “Biological function” are shown in the bar plot. The color of the bars indicates positive and negative enrichment scores as well as the corresponding log10 adjusted *p* values.

To capture the molecular effects of *TCF7L2* ablation, we performed RNA-seq experiments. Principal component analyses showed that HCT116 and HT29 *TCF7L2*^WT/Het^ and *TCF7L2*^KO^ clones separate well along principal component 1 which explains most of the variance (Fig. 2a). Applying an adjusted *p* value < 0.05 and an absolute value of log2 fold change (log2FC) > 0.5 as thresholds, we identified 2148 differentially expressed genes (DEGs) in HCT116 *TCF7L2*^KO^ versus *TCF7L2*^WT^ cells (Supplementary Table S3) and 3084 DEGs in HT29 *TCF7L2*^KO^ cells (Supplementary Table S4). Gene expression changes were mostly cell-line-specific. However, 630 DEGs were shared by HCT116 and HT29 *TCF7L2*^KO^ cells (Fig. 2b, Supplementary Table S5). Among these, 280 DEGs were upregulated and 122 DEGs were downregulated in both cellular backgrounds. Expression of the remaining 228 DEGs changed in opposite directions (Fig. 2b). Altogether, TCF7L2 appears to drive both specific and common gene expression programs in CRC cell lines.

To identify cellular processes possibly controlled by TCF7L2 across cell lines, we performed functional enrichment analyses of gene sets using Fisher’s exact test for the overlapping DEGs in HCT116 and HT29 *TCF7L2*^KO^ cells (Fig. 2c, Supplementary Table S6). According to these analyses, cell–cell and cell–matrix adhesion, migration, metabolic pathways, transcriptional regulation, and proliferation might be altered in *TCF7L2*^KO^ cells (Fig. 2c), the latter being reminiscent of the reduced population dynamics exhibited by HCT116 and HT29 *TCF7L2*^KO^ cells (see Fig. 1d), and the exhaustion of proliferating stem and progenitor cell compartments in *Tcf7l2*-deficient intestinal crypts [8, 10, 11]. Furthermore, Gene Ontology (GO) terms connected to Wnt signaling were among the features downregulated upon inactivation of *TCF7L2* (Supplementary Table S6). Consistent with this, expression of the Wnt target genes *MYC* and *TERT* [12–14, 24] was strongly decreased in HCT116 and HT29 *TCF7L2*^KO^ and *TCF7L2*^KOΔE6^ clones (Supplementary Fig. S9). Chromatin immunoprecipitation (ChIP) followed by quantitative PCR (qPCR) additionally demonstrated specific interactions of TCF7L2 with multiple regions at the *MYC* locus in *TCF7L2*^WT/Het^ but not in *TCF7L2*^KO^ cells (Supplementary Fig. S9e) [13, 14, 25–27]. Unfortunately, we could not detect TCF7L2 at a previously described TCF7L2-binding region at the *TERT* promoter (Supplementary Fig. S9f) [26, 27], albeit this does not rule out that TCF7L2 associates with other sites at the *TERT* locus. To further examine the impact of *TCF7L2* deficiency on Wnt/β-Catenin target gene expression, we extended our analyses to *AXIN2*, *ASCL2*, *EPHB3*, and *RNF43* [28–33]. However, these examinations were hampered by the fact that Wnt/β-Catenin targets are differentially expressed in CRC cells as reported by us and others [15, 34–37]. This likely explains why the comparatively low or undetectable expression of *AXIN2*, *ASCL2*, and *EPHB3* in HCT116 cells was not further affected by TCF7L2 inactivation (Supplementary Fig. S10). Yet, their expression decreased in *TCF7L2*-deficient HT29 *TCF7L2*^KO^ and *TCF7L2*^KOΔE6^ cells. Moreover, *RNF43* was consistently downregulated in both HCT116 and HT29 *TCF7L2*^KO^ and *TCF7L2*^KOΔE6^ cells (Supplementary Fig. S10). In agreement with the known function of TCF7L2, the results of the global and the targeted expression profiling in aggregate demonstrate that TCF7L2 deficiency vastly changes gene expression in HCT116 and HT29 cells and, as expected, impairs the transcriptional output of the Wnt/β-Catenin pathway.

### *TCF7L2* inactivation decreases proliferation

**Fig. 3:**
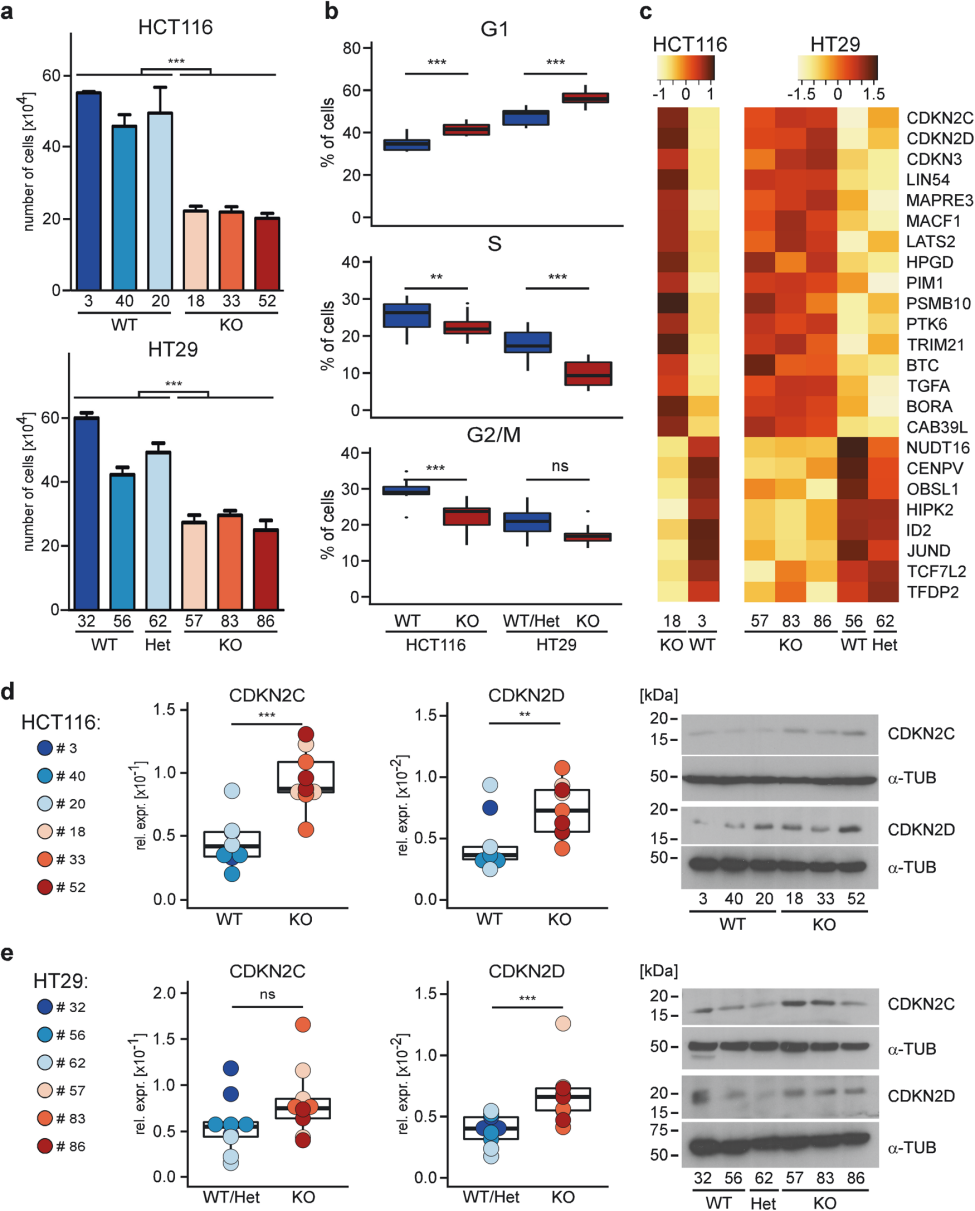
Loss of *TCF7L2* leads to reduced proliferation and delayed G1/S progression in CRC cells. **a** 1 × 10^4^ HCT116 cells and 5 × 10^4^ HT29 cells with the genotypes indicated were seeded, and incubated for 96 h. Then, the resulting cell counts were determined and displayed as bar plots. For statistical analysis, LMM was applied (*n* = 3). **b** To analyze differences in cell cycle distribution of *TCF7L2*^WT/Het^ and *TCF7L2*^KO^ cells, cells were stained with propidium iodide and analyzed by flow cytometry. The proportions of cells in different cell cycle phases are depicted by the box plots. For statistical analysis LMM was performed (*n* = 3). ns not significant. **c** The GO term “REGULATION_OF_CELL_CYCLE” was used to extract genes differentially expressed in *TCF7L2*^WT/Het^ and *TCF7L2*^KO^ cell clones and connected to cell cycle control. The *z*-scores of the *r*-log normalized counts of the genes were plotted as heatmap. **d**, **e** Expression of the cell cycle inhibitors *CDKN2C* and *CDKN2D* was analyzed in HCT116 (**d**) and HT29 cells (**e**) by qRT-PCR and western blotting. Colored dots represent qRT-PCR results for individual cell clones. Box plots summarize qRT-PCR results from all clones according to *TCF7L2* genotype. Data presented indicate *TCF7L2* expression relative to that of *GAPDH* (rel. expr.). For statistical analysis, LMM was applied (*n* = 3). In western blotting experiments, α-TUBULIN (α-TUB) served as loading control. Molecular weights are given in kDa. Representative results from one of three independent biological replicates are shown. ns not significant.

To experimentally address proliferative effects following loss of *TCF7L2*, we seeded the same numbers of *TCF7L2*^WT/Het^ and *TCF7L2*^KO^ and *TCF7L2*^KOΔE6^ cells and counted their progeny 96 h later. Both HCT116 and HT29 *TCF7L2*^KO^ and *TCF7L2*^KOΔE6^ cells showed significantly lower cell numbers compared with *TCF7L2*^WT/Het^ cells (Fig. 3a, Supplementary Fig. S11a, b). Flow cytometric analyses of DNA content further revealed increased G1 phase cell counts and decreased cell counts in S and G2/M phases in *TCF7L2*^KO^ and *TCF7L2*^KOΔE6^ clones (Fig. 3b, Supplementary Fig. S11c, d) suggesting impaired G1/S phase progression. To provide a molecular explanation for the observed cell cycle changes, we extracted DEGs from the GO term REGULATION_OF_CELL_CYCLE (Fig. 3c, Supplementary Table S6). These DEGs include *CDKN2C* and *CDKN2D* which negatively control the G1/S transition. By quantitative reverse transcription polymerase chain reaction (qRT-PCR) and western blotting we verified upregulation of *CDKN2C* and *CDKN2D* in *TCF7L2*^KO^ cells (Fig. 3d, e). Increased *CDKN2C* and *CDKN2D* transcript levels were also observed in HCT116 and HT29 *TCF7L2*^KOΔE6^ cells (Supplementary Fig. S11e, f). Altogether, TCF7L2 appears to promote proliferation of certain CRC cells like HCT116 and HT29 by suppressing cell cycle inhibitors while concomitantly stimulating the expression of mitogenic genes like *MYC*.

### Loss of TCF7L2 enhances migration and invasion of CRC cells

**Fig. 4:**
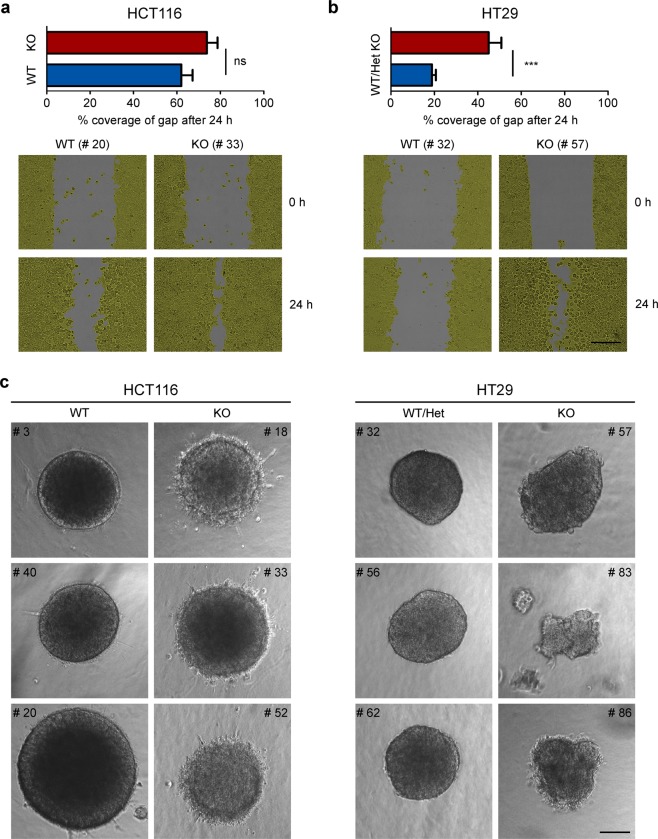
TCF7L2 deficiency enhances migration and invasion of CRC cells. **a**, **b** The migratory capacity of HCT116 (**a**) and HT29 cells (**b**) with WT and mutant *TCF7L2* genes was interrogated by wound-healing/gap closure assays. Bar graphs show the percent coverage of the gaps at 24 h after infliction. Shown are the combined mean values for all clones with the genotypes indicated. Error bars represent the SEM. Statistical analysis was performed using LMM (*n* = 4). Representative pictures of gaps at 0 and 24 h after wounding are shown in the lower panels. The scale bar represents 100 µm. **c** For three-dimensional invasion experiments, HCT116 and HT29 cells with WT and mutant *TCF7L2* genes were allowed to form spheres in hanging drops prior to embedding in a collagen matrix. Seventy-two hours after embedding, pictures of the spheroids were taken. Representative pictures from one of four independent biological replicates are shown for each clone. The scale bar represents 100 µm.

The RNA-seq results further indicated that *TCF7L2* affects cell migration and adhesion. Indeed, two-dimensional wound-healing assays showed that HCT116 and HT29 *TCF7L2*^KO^ cells are more migratory than *TCF7L2*^WT/Het^ cells (Fig. 4a, b, Supplementary Fig. S12). Likewise, motility of *TCF7L2*^KOΔE6^ cells was enhanced (Supplementary Fig. S11g, h). We also compared invasive properties of *TCF7L2*^KO^ and *TCF7L2*^WT/Het^ cells in a three-dimensional collagen I invasion assay (Fig. 4c). HCT116 *TCF7L2*^WT^ cells formed regularly shaped, round spheroids with smooth borders without evidence of invasion. In contrast, spheroids from HCT116 *TCF7L2*^KO^ clones exhibited irregular, rough surfaces caused by the single cell emigration, and sprouting of short strands of cells into the surrounding collagen matrix. The invasive behavior of HT29 *TCF7L2*^KO^ cells was less pronounced, but when compared with HT29 *TCF7L2*^WT/Het^ cells, *TCF7L2*^KO^ spheroids also presented with less regular and more raspberry-like shapes, and seemed to be less cohesive (Fig. 4c). We conclude that loss of TCF7L2 can promote migration and invasion of certain CRC cells.

### Loss of TCF7L2 increases adhesion of CRC cells to collagen I

**Fig. 5:**
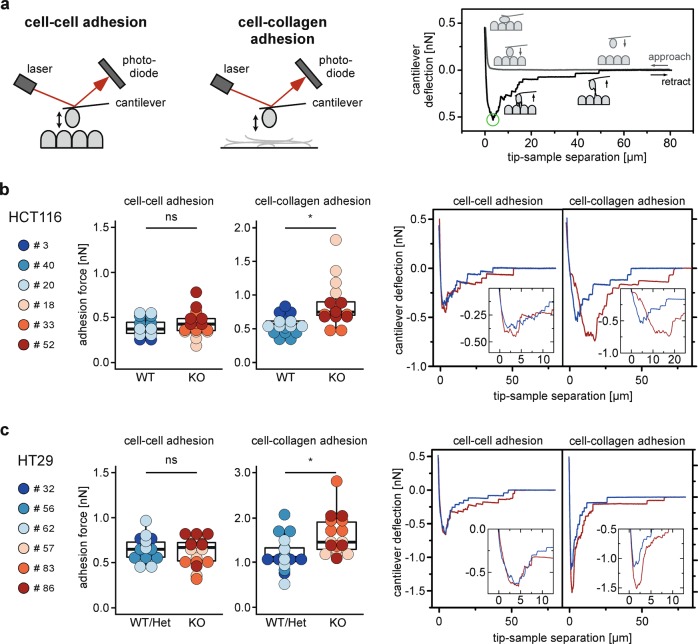
Cells display increased adhesion to collagen I upon loss of TCF7L2. **a** Left: scheme of the SCFS setup to measure cell–cell and cell–collagen adhesion. A cantilever with an immobilized cell is approached to substrates consisting of cell or collagen layers, and cantilever deflection is monitored via the reflection of a laser into a 4-quadrant photodiode. Cantilever bending is directly proportional to the force acting on the cantilever. Right: schematic force curves. During approach (gray curve) the cantilever deflection remains at 0 pN until contact with the sample is reached. Upon further approach the cantilever bends upward exerting force on the contact until the defined force set point is reached, and held for a defined timespan. Upon retraction (black curve) the contact force is continuously released until further retraction leads to downward bending as the cantilever starts pulling on the bonds that were formed during contact. The cell(s) and bonds are stretched until a critical value is reached, termed detachment force, *F*_det_ (green circle), at which the bonds start to successively disengage until the cell is completely freed and the retraction curve arrives at the level of the approach curve. Statistical analyses of high numbers of retract curves from multiple cells yield information about the strength of cell–substrate adhesion. HCT116 (**b**) and HT29 cells (**c**) with WT und mutant *TCF7L2* genes were measured by SCFS to quantify cell–cell and cell–collagen adhesion as indicated. The cell attached to the cantilever originated from the same clone as seeded on the coverslip. At least five cells were measured per cell clone and 30 force curves were recorded per cell. Dots represent the mean of the maximum adhesion force of each cell. Dot color identifies individual cell clones, and the box plot summarizes data according to *TCF7L2* genotype. For statistical analysis, LMM was applied. ns not significant. Representative force curves for both experimental setups are shown on the right. WT curves blue, KO curves red. Inserts in graphs show blow ups of the force curves around *F*_det_.

Next, we investigated whether cell–cell adhesion might be impaired by loss of TCF7L2. To address this, we used single-cell force spectroscopy (SCFS) which is based on atomic force microscopy and facilitates the quantification of cell–cell and cell–substrate interactions at a single cell level (Fig. 5a). Surprisingly, for both, HCT116 and HT29 cells, there was no difference in intercellular adhesion forces between *TCF7L2*^WT/Het^ and *TCF7L2*^KO^ cells (Fig. 5b, c). However, *TCF7L2*-deficient HCT116 and HT29 cell derivatives displayed enhanced adhesion to collagen I (Fig. 5b, c), which fits to the altered appearance and sprouting behavior of *TCF7L2*^KO^ spheroids. Thus, TCF7L2 not only controls cell proliferation and migration, but also interactions of HCT116 and HT29 CRC cells with extracellular matrix components.

### Loss of *TCF7L2* disturbs a gene-regulatory network related to cell adhesion and migration

**Fig. 6:**
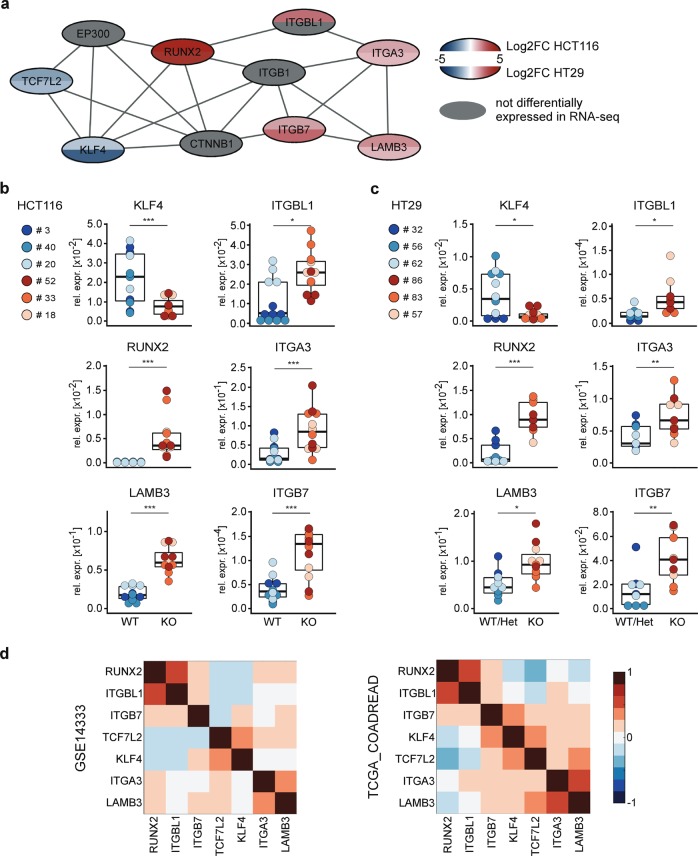
*TCF7L2* deficiency disturbs a molecular network connected to migration and adhesion. **a** The string database was used to detect a regulatory network comprising several transcription factors and cell adhesion molecules. Shading of the nodes represents the log2FC of gene expression in the respective cell line. Gray nodes denote genes that are not differentially expressed in the RNA-seq datasets. **b**, **c** Expression of components of the network shown in (**a**) was analyzed by qRT-PCR in HCT116 (**b**) and HT29 cells (**c**) with WT and mutant *TCF7L2* genes. Expression was normalized to *GAPDH* and is shown as relative expression (rel. expr.). Colored dots represent results from independent biological replicates for the different cell clones. The box plots summarize data according to the *TCF7L2* genotype. For statistical analysis, LMM was applied (*n* = 4). **d** Pairwise correlation analyses of the genes indicated based on the publicly available gene expression datasets for human CRC samples (GSE14333: 290 samples; TCGA_COADREAD: 212 samples). Red and blue color shading (scaling indicated by the colored bar) represents the Pearson correlation efficient for correlated and anticorrelated expression, respectively.

To provide a mechanistic framework for the migration/invasion-related phenotypic alterations of *TCF7L2*-deficient cells we integrated our RNA-seq results with a query of the string database [38]. This identified a molecular network that linked *TCF7L2* among others to the *KLF4* and *RUNX2* transcription factor genes, the laminin subunit *LAMB3*, and the integrin genes *ITGA3*, *ITGB7*, and *ITGBL1* (Fig. 6a). KLF4 suppresses CRC cell migration and invasion [39–41], whereas RUNX2 and its target gene *ITGBL1* are pro-migratory and pro-metastatic [42–45]. Similarly, overexpression of *ITGA3* and *ITGB7* augments migration, matrix adhesion, and metastasis formation [46, 47]. ITGA3 additionally mediates collagen adhesion [48]. Interestingly, ITGA3 marks numerous cell protrusions in HCT116 *TCF7L2*^KO^ cells, which are not detected in *TCF7L2*^WT^ cells (Supplementary Fig. S13). We additionally confirmed that *KLF4* and *RUNX2* were down- and upregulated, respectively, in HCT116 and HT29 *TCF7L2*^KO^ and *TCF7L2*^KOΔE6^ cells (Fig. 6b, c, Supplementary Figs. S14–S17). Furthermore, compared with *TCF7L2*^WT^ cells, expression of *LAMB3*, *ITGBL1*, *ITGA3*, and *ITGB7* was elevated in *TCF7L2*-deficient cells (Fig. 6b, c, Supplementary Figs. S14, S17), except for *ITGBL1*, *ITGA3*, and *ITGB7* whose transcript levels were largely unchanged in HT29 *TCF7L2*^KOΔE6^ cells (Supplementary Fig. S17). Possibly, TCF7L2 affects these genes only indirectly through intermediate regulatory steps which appear to be subject to clonal variation. We therefore focused on the robustly deregulated transcription factor genes *KLF4* and *RUNX2*, and investigated whether TCF7L2 directly regulates them. To identify potential TCF7L2-binding sites at the *KLF4* and *RUNX2* loci, we explored existing TCF7L2 ChIP-seq datasets [26, 27], which suggested the presence of one promoter-proximal and two intragenic TCF7L2-binding regions at the *KLF4* and *RUNX2* loci, respectively (Supplementary Fig. S18). By ChIP-qPCR, however, we did not detect specific occupancy of the *KLF4* promoter region by TCF7L2 (Supplementary Fig. S18a). In contrast, TCF7L2 associated with both *RUNX2* intragenic elements in *TCF7L2*^WT^ cells but not in *TCF7L2*^KO^ cells (Supplementary Fig. S18b), thereby identifying *RUNX2* as a direct target gene that is repressed by TCF7L2 in HCT116 and HT29 CRC cells.

To assess whether the regulatory relationships among TCF7L2, KLF4, RUNX2 and the cell adhesion factors observed in CRC cell lines might be relevant for human CRC samples, we performed pairwise correlation analyses based on publicly available transcriptome data. In two different CRC cohorts, expression of *RUNX2* and *ITGBL1* was strongly positively correlated (Fig. 6d). This is consistent with the known direct control of *ITGBL1* by RUNX2 and supports the significance of the analyses. Matching the results of our expression analyses in CRC cell lines, expression of *TCF7L2* was anticorrelated to that of *RUNX2* and *ITGBL1* in CRC transcriptomes (Fig. 6d), whereas a positive correlation existed for expression of *TCF7L2* and *KLF4*. The relationships between expression of *TCF7L2* and *RUNX2* and their potential targets *ITGA3*, *ITGB7*, and *LAMB3* were more ambiguous, hinting at indirect and more complex connectivity, possibly depending on the precise cellular context. Nonetheless, the combined results of the expression analyses in cell lines and tumors suggest that loss of TCF7L2 can suppress a regulatory network that otherwise might mobilize CRC cells and thereby promote cancer cell dissemination.

### The frequency of *TCF7L2* mutations/copy number loss is positively correlated with increased tissue invasion and metastasis in colon adenomacarcinomas

**Fig. 7:**
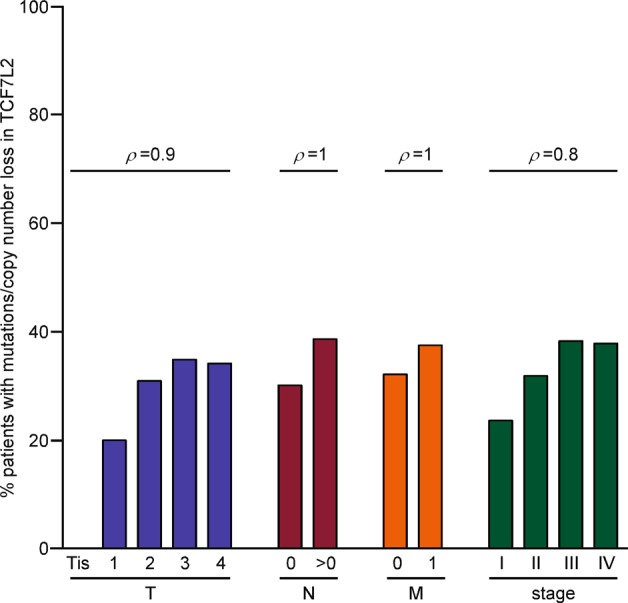
The frequency of *TCF7L2* mutations/copy number loss increases during tumor progression. The percentages of colon adenocarcinoma patients with *TCF7L2* mutations/copy number loss in relation to different categories according to TNM and AJCC staging systems were computed and plotted as indicated. Spearman’s rank correlation analysis was used to examine whether the frequency of *TCF7L2* mutations/copy number loss follows an increasing order in parallel with tumor progression. The individual Spearman’s rank correlation values (*ρ*) for all categories are shown above the bars. The mean of the correlation values across all groups (TNM and AJCC staging) is 0.925 (*p* value = 0.0003028 [95% CI 0.772–1.077]). Tis: carcinoma in situ. Increasing *T* ratings indicate tumor spread to the submucosa (*T* = 1) and beyond (*T* ≥ 2). *N* denotes absence (*N* = 0) or presence (*N* > 0) of lymph node metastases. *M* indicates absence (*M* = 0) or presence (*M* = 1) of distant organ metastases.

The observed changes in CRC cell motility and invasiveness following inactivation of *TCF7L2* prompted us to analyze potential relationships between the occurrence of *TCF7L2* genomic alterations and colorectal tumor progression using publicly available data for colon adenocarcinoma (COAD) from The Cancer Genome Atlas (TCGA) collection. Upon stratification according to the TNM and AJCC staging systems, the percentages of patients with inactivating mutations/copy number loss in *TCF7L2* turned out to be higher among tumors which had locally invaded to the submucosa and beyond (*T* rating > 1; stages I/II), as well as spread to the lymph nodes (*N* ≥ 1; stage III) or to distant organs (*M* = 1; stage IV) (Fig. 7, Supplementary Table S7). Thus, the frequency of *TCF7L2* mutations/copy number loss are positively correlated with increased tissue invasion and metastasis which is consistent with a migration and invasion suppressor role for *TCF7L2* in colorectal tumors.

## Discussion

Conflicting observations concerning the role of *TCF7L2* in colorectal carcinogenesis prompted us to systematically investigate *TCF7L2* dependence/independence of CRC cells and the phenotypic consequences of *TCF7L2* inactivation. As key findings we present that *TCF7L2*-deficient CRC cells are viable and proliferate, thereby revealing a fundamental change in the functional importance of *TCF7L2* compared with the healthy intestinal epithelium. In addition, *TCF7L2* loss-of-function enhanced motility and invasiveness, two traits associated with increased malignancy. Thus, our data suggest that in certain CRC contexts *TCF7L2* may function as invasion suppressor which provides an explanation for the observed frequency of TCF7L2 mutations.

The spectrum of mutations in *TCF7L2* in CRC encompasses single nucleotide exchanges and small insertions/deletions leading to nonsense, missense, and frame-shift mutations throughout the entire coding region, as well as translocations, copy number changes and deep deletions, many of which are likely to result in alleles with partial or complete *TCF7L2* loss-of-function [2, 17, 18]. As a caveat, the available genomic data and information about variant allele frequencies do not reveal whether a given tumor sample harbors one or two defective *TCF7L2* alleles. In contrast, our study showcases the consequences of fully inactivated *TCF7L2* and complete absence of TCF7L2 protein isoforms comparable to genetically engineered mouse models [8, 10, 11]. Thus, it is possible that colorectal tumors retaining one functional allele or expressing mutant TCF7L2 protein variants exhibit phenotypes different from *TCF7L2*^KO^ and *TCF7L2*^KOΔE6^ cells.

By disrupting or deleting *TCF7L2* exon 6 we readily achieved biallelic *TCF7L2* inactivation in LoVo, HCT116, and HT29 cells. Likewise, SW480 cells tolerate KO of *TCF7L2* [11]. These four CRC cell lines harbor various combinations of oncogenic lesions in the *APC*, *CTNNB1*, *KRAS*, *BRAF*, *PIK3CA*, *TP53*, *SMAD4*, and *TGFBR2* genes [21], and represent different CRC subtypes [22, 23]. Thus, dispensability of *TCF7L2* in CRC cells does not seem to be rare, but the mechanisms that allow CRC cells to acquire *TCF7L2* independence do not become immediately apparent. According to one model, redundancy among TCF/LEF family members allows TCF7, TCF7L1, and LEF1 individually or collectively to substitute for TCF7L2 [11]. Indeed, inactivation of *TCF7L2* in different cellular backgrounds came along with graded phenotypic changes, which inversely correlated with the range of TCF/LEF family members present. However, this could be a mere coincidence. For instance, TCF7 and TCF7L2 are coexpressed in the healthy intestinal epithelium, yet, TCF7 fails to rescue *TCF7L2* inactivation. Likewise, the absence of the C-clamp domain from TCF7L1 and LEF1, as well as functional differences and even antagonism among TCF7L1, LEF1, and TCF7L2 [3–5, 20, 36, 49] suggest that other TCF/LEF family members possess only a limited potential to functionally replace TCF7L2. Alternative explanations for the survival of *TCF7L2*-deficient CRC cells, and the differential penetrance of the *TCF7L2* loss-of-function phenotype could be that CRC cells in general appear to be variably dependent on TCF/LEF activity and even Wnt/β-Catenin pathway function [37, 50].

Deletion of *TCF7L2* produced massive changes in gene expression and cellular behavior. Thus, unlike previously claimed [11], the absence of TCF7L2 in CRC cells is not phenotypically neutral. However, biallelic inactivation of *TCF7L2* was necessary to bring out phenotypic changes, and no evidence for haploinsufficiency was obtained [9]. Our findings of decreased proliferation and G1/S cell cycle delay in the absence of TCF7L2 are completely concordant with substantial evidence connecting TCF7L2 to the control of cell division in the healthy intestinal epithelium and in CRC cells [8, 10–12, 37]. Fittingly, TCF7L2 stimulates the expression of several genes that promote cell cycle progression [12, 51, 52]. Based on our findings, TCF7L2 additionally suppresses cell cycle inhibitors. Thus, it appears that the role of TCF7L2 as positive regulator of proliferation in healthy intestinal cells is preserved in a CRC context. However, the exclusive reliance of intestinal stem and progenitor cells on mitogenic Wnt/β-Catenin signaling appears to be lost in the course of colorectal tumorigenesis.

The proliferation-stimulating function of TCF7L2 in CRC cells observed here disagrees with previous findings [19]. However, Tang et al. assessed proliferation only indirectly through a metabolic assay, which could be affected by *TCF7L2* deficiency irrespective of any proliferative effects. Notably, TCF7L2 was linked to glycolysis [53, 54]. Likewise, our RNA-seq data imply that TCF7L2 influences cellular metabolism. Therefore, it is possible that the consequences of TCF7L2 knockdown in earlier work were largely misinterpreted.

Alongside with impaired proliferation, *TCF7L2*-deficient cells exhibited morphological changes, increased motility, invasion, and adhesion to collagen I. In line with this, loss of TCF7L2 was accompanied by a switch in transcription factor expression from migration and invasion-suppressing KLF4 to pro-migratory and pro-metastatic RUNX2. This was paralleled by the upregulation of *LAMB3* and several integrins, although integrin deregulation was observed more robustly in HCT116 cells. This might be due to clonal variability in the expression of one or more intermediate regulators that connect TCF7L2 to the integrin genes, but could also hint that additional gene expression changes contribute to the more migratory and invasive phenotype following *TCF7L2* ablation in a cell-type-specific manner. The cell guidance gene *EPHB3* [32] which was downregulated specifically in HT29 *TCF7L2*^KO^ and *TCF7L2*^KOΔE6^ cells, could be an example. Thus, the full range of genes related to cell–cell and cell–matrix adhesion under control of TCF7L2, KLF4, and RUNX2, and the precise regulatory relationships among these factors remain to be determined. In this regard, we already identified *RUNX2* as a novel TCF7L2 target gene. Altogether, the observed gene expression changes provide a plausible molecular explanation for the increased migratory and invasive capacity of *TCF7L2*-deficient CRC cells, and mark *TCF7L2* as an invasion suppressor gene.

Apparently, TCF7L2 controls highly divergent gene expression programs in CRC cells. Cell-line-specific TCF7L2-dependent gene expression patterns could represent different subprograms of the overall transcriptome whereby Wnt/β-Catenin signaling controls proliferation, lineage decisions, positioning, differentiation, and maturation of intestinal epithelial cells [1]. These subprograms might segregate in cancer cells due to epigenetic inactivation [55, 56] or because of variations in the activity of cooperating signal transduction pathways [35, 37, 57, 58].

In summary, the physiological functions of TCF7L2 appear to be largely retained during colorectal carcinogenesis: control of proliferation and motility of CRC cells precisely reflect the roles of Wnt/β-Catenin signaling and its nuclear effector TCF7L2 in the healthy intestinal epithelium where they stimulate proliferation of stem and progenitor cells, and control cell migration and allocation along the crypt axis [8, 10, 11, 32, 51]. However, whereas TCF7L2 is essential in noncancerous tissue, it is not required for CRC cell viability. The widespread dispensability of *TCF7L2* combined with its traits of an invasion suppressor shed new light on the role of *TCF7L2* in CRC cells, and are highly relevant for the assessment and potential selection of β-Catenin/TCF7L2 complexes as targets for therapeutic intervention in CRC.

## Material and methods

### Cell lines, oligonucleotides, and antibodies

Cell lines used and their culture conditions are presented in Supplementary Table S8. Oligonucleotides for ChIP-qPCR/qRT-PCR, and antibodies for immunodetection experiments are listed in Supplementary Tables S9 and S10, respectively.

### Intestinal organoid culture and fractionation of the small intestine

The KO cassette was flipped out [59, 60] of the genome of *C57BL/6NTac-Tcf7l2*^*tm1a(EUCOMM)Wtsi*^*/WtsiIeg* mice (European Mouse Mutant Archive strain EM:07858), and offspring retaining the floxed *Tcf7l2* exon 6 was bred to C57BL/6N-*tgVillinCreERT2* animals [61]. Five females (18–26 weeks old) from the resulting C57BL/6N-*Tcf7l2*^*flox/flox*^; *tgVillinCreERT2* mice were used to generate intestinal organoids as described [62], except that colon organoids additionally received 100 ng/ml murine Wnt3a (Peptrotech, Rocky Hill, NJ, USA). Mice were handled in accordance with legal regulations at the Center for Experimental Models and Transgenic Service of the University of Freiburg Medical Center (project registration number: X-17/07S). To delete *Tcf7l2* exon 6, organoids were treated with 0.5 µM 4-hydroxytamoxifen for 24 h. The small intestinal epithelium was separated into crypt and villus fractions as previously described [35].

### Genome editing

CRISPR/Cas9-mediated genome editing was performed as before [63], except that gRNAs were cloned into pMuLE_ENTR_U6_stuffer_sgRNA_scaffold_L1-R5 [64]. Target sequences of gRNAs were: 5′-GTTTTGGGGTCTACGTCGGC-3′ (*TCF7L2* exon 6; GRCh38:10:113,141,184-113,141,316); 5′-GGCTGCTGGAACCGGCTTGA-3′ and 5′-ACGGAGCCAAGGTAGAGTGC-3′ (located in *TCF7L2* introns 5 and 6). 1 × 10^6^ cells were nucleofected with 600 ng of each gRNA construct, and 800 ng of Cas9-turboRFP or Cas9-GFP expression vectors. RFP^+^/GFP^+^ cells were single cell sorted into 96 well plates 72 h after nucleofection, expanded, and screened for TCF7L2 expression by western blotting (HCT116 and HT29 *TCF7L2*^KO^ cells), or for exon 6 deletion by PCR (LoVo *TCF7L2*^KOΔE6^ cells, HCT116 and HT29 *TCF7L2*^KOΔE6^). Details about the genomic state of the *TCF7L2* locus for all cell clones used in this study can be found in Supplementary Table S2.

### Western blotting

Except for the experiments shown in Supplementary Fig. S7, for which whole cell lysates made with RIPA buffer were employed [63], we investigated TCF/LEF expression using nuclear extracts which were obtained following a published protocol [35], albeit without dounce homogenizing for disrupting the cells. All other antigens were analyzed in whole cell lysates which were prepared as before [35]. For antigen detection, cell lysates and nuclear extracts were separated by SDS-PAGE and further processed by western blotting as described [35].

### Immunofluorescence and immunohistochemistry

Immunofluorescence staining of cultured cells was carried out as described [65] using 2 × 10^5^ cells seeded on glass slides coated with 0.1% gelatin. For immunohistochemical analyses of mouse tissue, the gut of healthy C57BL/6N mice was isolated and washed with PBS prior to fixation in 4% PFA. The tissue was embedded in paraffin, cut (5–8 µm), deparaffinized, and antigens were retrieved by boiling in 0.01 M citrate buffer. After blocking endogenous peroxidase in 10% (v/v) methanol, 3% H_2_O_2_ (v/v) in H_2_O for 20 min at room temperature, the slides were washed once in PBS-Tween 20, and the sections were encircled by PAP pen. The subsequent staining procedure was performed using the Vectastain Elite ABC Peroxidase kit (#VEC-PK-6100, BIOZOL, Eching, Germany) following the manufacturer’s protocol with minor modifications: the blocking time was extended to 30 min, primary antibody incubation to overnight at 4 °C, and secondary antibody incubation to 1 h. DAB solution was used as peroxidase substrate solution. For counterstaining, the tissue sections were incubated for 5 min in filtrated Mayer’s hemalum solution (Merck, Darmstadt, Germany), rinsed with water for 15 min, and mounted. Formaldehyde-fixed and paraffin-embedded tissue specimens from patients with CRC who had undergone tumor surgery at the University of Regensburg between 2006 and 2018 for this diagnosis, were included in a retrospective histological analysis of tumor tissue and adjacent healthy colon tissue. Patients gave written informed consent and the study was performed according to the principles of Helsinki and approved by the Local Ethics Committee (No. 14-101-0014). Staining was performed as previously described [66]. A tissue microarray containing 90 adenocarcinoma tissue specimens with matched cancer-adjacent tissue or adjacent normal tissue (#CO1801, US Biomax Inc., Derwood, MD, USA) was processed and stained the same way.

### Correlation analysis for TCF7L2 mutations/copy number loss and tumor staging

We extracted information about clinical parameters, somatic single nucleotide variations (SNV) and somatic copy number changes for a total of 351 patients from the TCGA COAD collection (https://portal.gdc.cancer.gov; date of download: November 5, 2019). In case of SNVs, we considered missense mutations, nonsense mutations, splice site alterations, and frame shift-causing insertions and deletions. The percentages of patients with *TCF7L2* mutations/copy number loss for the different categories of the TNM and AJCC staging systems was computed, and plotted in the order of increasing ratings for each category. To examine whether the frequency of *TCF7L2* mutations/copy number loss is positively correlated with increased TNM and AJCC staging, Spearman’s rank correlation analyses were performed as follows: for each group of TNM categories/AJCC stages, the correlation value was calculated between the percentages of the patients with *TCF7L2* mutations/copy number loss from the different categories/stages and a predefined numerical vector. The numerical vectors are “1, 2, 3, 4, 5” for the T categories, “1, 2” for the N and M categories, and “1, 2, 3, 4” for the AJCC stages, thereby supporting strictly ascending orders. After computing the individual correlation values, a *t* test was used to calculate the average of the correlation values (alternative hypothesis: true = mean of the correlation values is not equal to 0).

### Flow cytometry

3 × 10^5^ cells/well were seeded in six-well plates and incubated for 48 h. Cells were then processed for flow cytometry as described [63]. BD FACSCalibur (BD Biosciences, Heidelberg, Germany) and CytoFLEX machines (Beckman Coulter, Krefeld, Germany) together with the FlowJo software were used for analysis.

### Work with RNA and RNA-seq data analysis

RNA isolation, cDNA synthesis, and qRT-PCR were performed as described [63], except that a cDNA amount equivalent to 20 ng total RNA was used for qRT-PCR. For transcriptome analyses, extracted RNA was paired-end sequenced on an Illumina HiSeq4000 at the Genome and Proteome Core Facility of the German Cancer Research Center, Heidelberg, Germany. Paired-end reads were filtered using Trimmomatic [67]. Selected reads were aligned to the human reference genome GRCh37. Read counts per gene were quantified with STAR aligner [68]. After excluding genes with low expression levels, 23,209 and 23,030 genes were considered for further processing for HCT116 and HT29 cells, respectively. For HT29, the two replicates for each of the five clones were integrated by averaging the corresponding number of counts. The differential expression analysis is based on a generalized linear model with a negative binomial distribution (DESeq2) [69]. For each cell line, *TCF7L2*^KO^ cells were compared with *TCF7L2*^WT/Het^ cells. The 2148 and 3084 top DEGs for HCT116 and HT29 cells, respectively, were selected by applying a Benjamini–Hochberg false discovery rate-corrected *p* value < 0.05 and absolute values of log2FC > 0.5. From these sets of genes we determined the intersection of the significantly DEGs across the two cell lines. For functional enrichment analyses of gene sets, we applied a Fisher’s exact test [70] to the selected top DEGs from each cell line, and to the 402 overlapping genes that are deregulated in both cell lines. For this, the stats R package was employed using a related set of background genes and the GO term collection “Biological Processes” as annotation. For the cell line-specific analyses, the 23,209 (HCT116 cells) and the 23,030 (HT29 cells) genes identified by applying the DESeq2 package, were taken as the background. For the overlapping gene set, the union of the top DEGs in both cell lines (i.e., 4602 genes) was taken as background. We calculated enriched GO terms for up- and downregulated genes separately, and retained GO terms with a significant adjusted *p* value of < 0.05 for both the cell line-specific calculations and the overlapped genes. RNA-seq data were deposited at the GEO database under accession number GSE135328.

### ChIP

Chromatin isolation and ChIP-qPCR were performed as before [35], except that nuclei were isolated by NEXSON [71]. For each ChIP, 100 µg of Chromatin and 20 µl of TCF7L2 antibody were used.

### Cell migration and invasion

Cell migration assays were carried out as before [63] using 5 × 10^4^ cells per chamber of ibidi^®^ cell culture inserts (#81176 ibidi GmbH, Martinsried, Germany). Three-dimensional growth of cancer cell spheroids in collagen I and invasion of cells into the collagen matrix was analyzed as described [72]. Pictures of the spheroids were taken 72 h after embedding.

### Single-cell force spectroscopy

SCFS was carried out on a JPK NanoWizard III/CellHesion200 device (JPK Instruments AG, Berlin, Germany) mounted on a Nikon inverted light microscope with a petri dish heater. Measurements were done in 35 mm petri dishes (TPP, Sigma-Aldrich, Taufkirchen, Germany) in CO_2_-independent medium (ThermoFisher Scientific, Dreieich, Germany) at 37 °C. Tipless cantilevers (MLCT-O10, Bruker, Bremen, Germany) were calibrated using the thermal noise method, and functionalized with mannose-binding concanavalin A using standard procedures [73]. Coverslips (9 mm Ø) were coated with cells or collagen I, and placed into 35 mm Ø petri dishes. For attachment of a single cell to the very front of the cantilever, a cell was “fished” from a droplet of cell suspension that was pipetted into the petri dish far away from the coated coverslips. Force curves were recorded using an approach/retract speed of 20 or 30 μm/s depending on cell type and substrate. Contact was held at a constant force of 0.50 nN for 1.5 s (HT29 cell–cell and cell–collagen contact, HCT116 cell–collagen contact) or 0.25 s (HCT116 cell–cell contact). Five to seven cells were probed per condition for each cell clone, and 30 approach-and-retraction cycles per cell were quantitatively evaluated using the JPK SPM data processing software.

### Query of the STRING database and publicly available transcriptome data

A list comprising *TCF7L2, RUNX2, KLF4, ITGA3, ITGBL1*, and *ITGB7* was submitted to the string database (state: January 2019) for network analysis using default settings and a single expansion step [38]. TCF/LEF expression data in human normal tissue und CRC samples were extracted from a publicly available microarray dataset [74], and the mean of the log2 expression values for genes-of-interest in the different samples was calculated using GraphPad Prism. The TCGA_COADREAD dataset was analyzed using cBioPortal (https://www.cbioportal.org/) [75, 76]. TCGA_COADREAD datasets containing gene expression information for human CRC samples (https://xenabrowser.net/) and the GSE14333 dataset were used for pairwise correlation analysis as described [35].

### Statistical analysis

Statistical analysis was performed using a linear mixed model (LMM) to account for random and fixed effects. Normal distribution of data was assessed using the car R package [77]. For LMM analysis, the lme4 R package was applied [78]. The ggplot2 R package [79] was used to generate box plots depicting the median, the lower, and upper quartile. Whiskers represent 1.5 times the interquartile range. The *p* values for significant changes are represented as follows: *p < 0.05; **p < 0.01; ***p < 0.001. For each series of experiments the corresponding number (*n*) of independent biological replicates is given in the figure legends.

## Supplementary information

Supplementary Figures_S1-S18

Supplementary table S1

Supplementary table S2

Supplementary table S3

Supplementary table S4

Supplementary table S5

Supplementary table S6

Supplementary table S7

Supplementary table S8

Supplementary table S9

Supplementary table S10
