# Supplementary material for: Loss of the nuclear Wnt pathway effector TCF7L2 promotes migration and invasion of human colorectal cancer cells
Source: Oncogene. 2020 Mar 20;39(19):3893–909. doi: 10.1038/s41388-020-1259-7 (PMC7203011; doi:10.1038/s41388-020-1259-7)
Supplement: Supplementary file 1 — Supplementary Figures_S1-S18 [file 41388_2020_1259_MOESM1_ESM.pdf]

Supplementary Figures

Loss of the nuclear Wnt pathway effector TCF7L2 promotes migration and invasion of human colorectal cancer cells

Janna Wenzel, Katja Rose, Elham Bavafaye Haghighi, Constanze Lamprecht, Gilles Rauen, Vivien Freihen, Rebecca Kesselring, Melanie Boerries, and Andreas Hecht

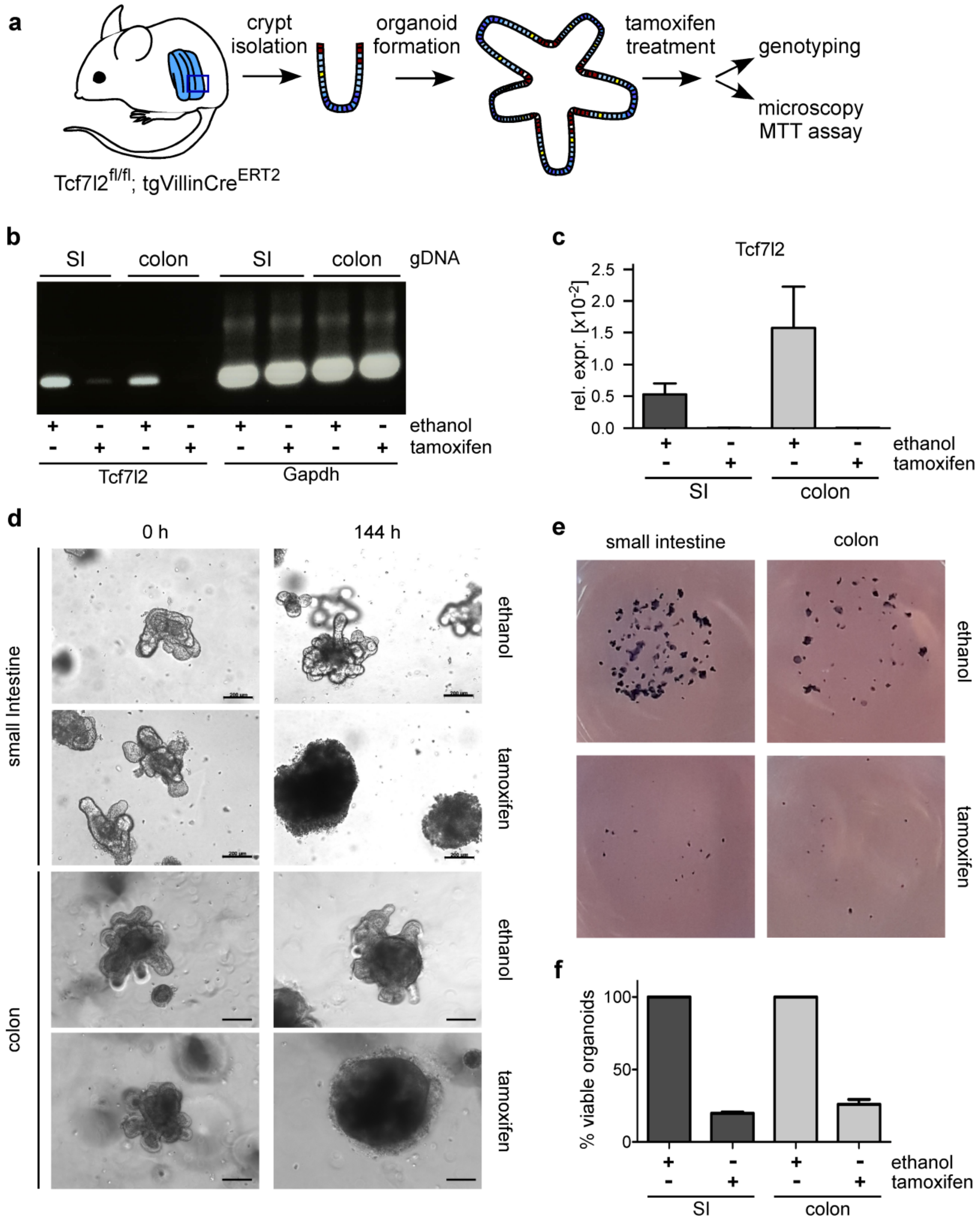

Supplementary Fig. S1 Tcf7l2 is essential for the survival of mouse intestinal organoids. **a** Experimental outline for the preparation and analyses of intestinal organoids. Crypts from the small intestine (SI) and colon of *Tcf7l2*$^{fl/fl}$; *tgVillinCre*^{ERT2} mice were isolated and cultured for organoid formation. To induce recombination of *Tcf7l2* exon 6, organoids were treated with 0.5 µM tamoxifen or ethanol as solvent control for 24 h. Thereafter, organoid cultures were split for separate genotyping and phenotypic analyses by microscopy and MTT staining. **b** Genomic DNA (gDNA) from SI and colon organoids treated with ethanol or tamoxifen for 24 h was subjected to PCR with primers flanking exon 6 of the *Tcf7l2* gene. Parallel PCRs with primers for the housekeeping gene *Gapdh* served as control to assure presence and integrity of genomic DNA. One representative result from four independent biological replicates is shown. **c** Expression of *Tcf7l2* was analyzed by qRT-PCR upon treatment of organoids from the small intestine (SI) and colon of *Tcf7l2*$^{fl/fl}$; *tgVillinCre*^{ERT2} mice with ethanol or tamoxifen for 24 h. Transcript levels are presented as relative expression (rel. expr.) normalized to those of *Gapdh*. Mean values and the standard errors of the mean (SEM) are displayed. (n=3). **d** Microscopy pictures of SI and colon organoids taken immediately after treatment with ethanol or

tamoxifen (0 h) and 144 h later. Representative images from one of four independent biological replicates are shown. The scale bars represent 200 µm. **e** SI and colon organoids from *Tcf7l2*$^{fl/fl}$; *tgVillinCre*^{ERT2} mice were pretreated with 0.5 µM tamoxifen or ethanol as solvent control for 24 h. Then, the medium was exchanged and the organoids were cultured for an additional 144 h before MTT staining was performed to visualize living cells. Representative images from one of three independent biological replicates are shown. **f** Viable organoids from cultures as shown in (e) were counted, and the percentage of viable organoids compared to the ethanol-treated control samples was calculated. The bars represent the mean and the corresponding SEM (n=3).

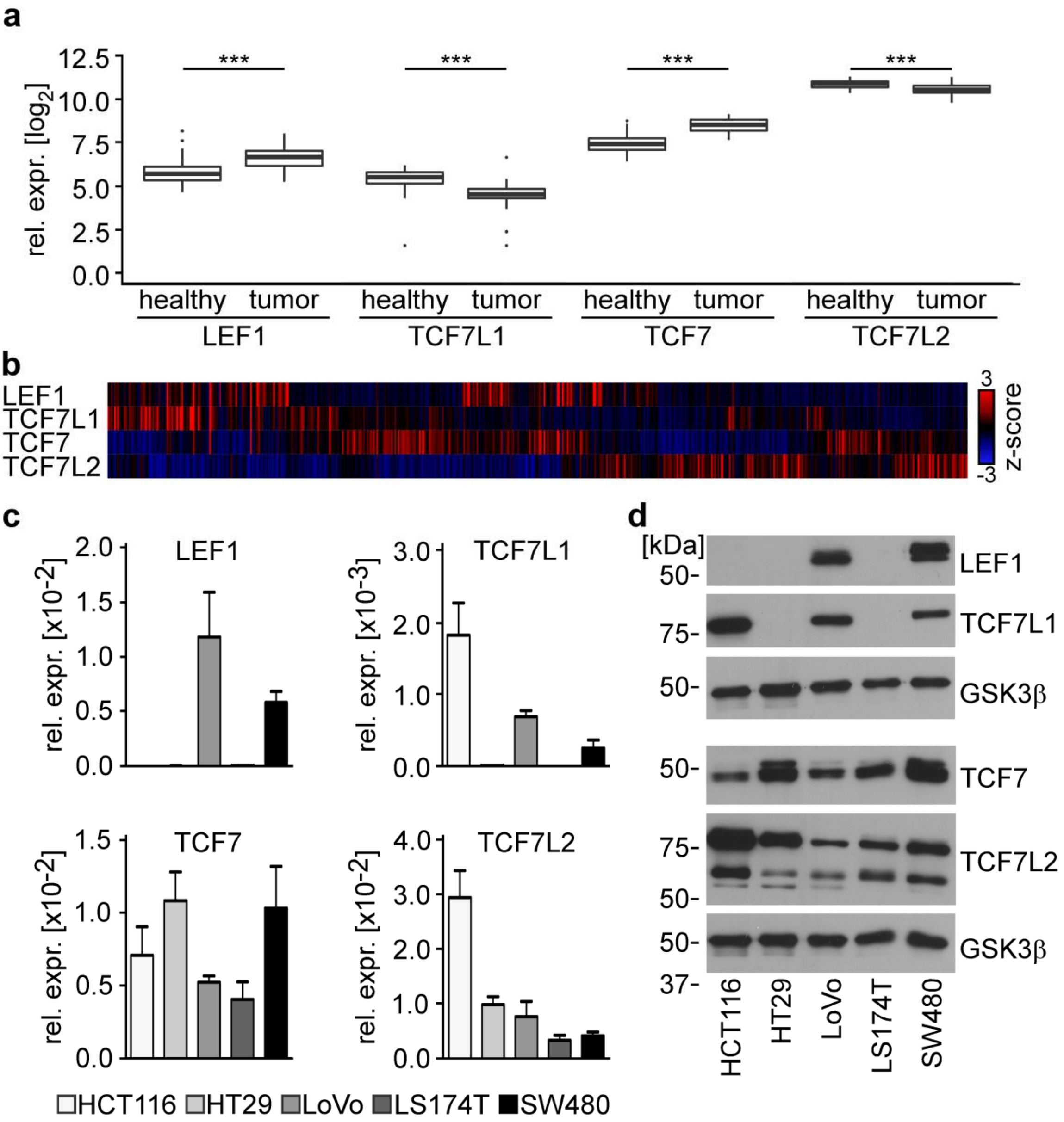

Supplementary Fig. S2 Expression patterns of TCF/LEF family members are variable in CRC specimens and differ from the healthy epithelium. **a** Comparative expression analyses of TCF/LEF family members in publicly available transcriptome datasets from 32 matched healthy and tumor samples of CRC patients. Statistical analysis was performed using Mann Whitney test. **b** Expression patterns of TCF/LEF family members in 212 human CRC specimens from the TCGA-COADREAD data collection were compiled using cBioPortal. The z-scores of the relative expression levels are depicted by the red/blue color gradient. **c** The expression patterns of the TCF/LEF family members were analyzed by qRT-PCR in five CRC cell lines. Transcript levels are presented as relative expression (rel. expr.) normalized to those of *GAPDH*. Mean values and the SEM are displayed. (n≥4). **d** Nuclear extracts were generated from the indicated CRC cell lines and used for Western blot analysis to assess protein abundance of TCF/LEF family members. GSK3β was used as loading control. Molecular weights are given in kDa. Representative results from one of three independent biological replicates are shown.

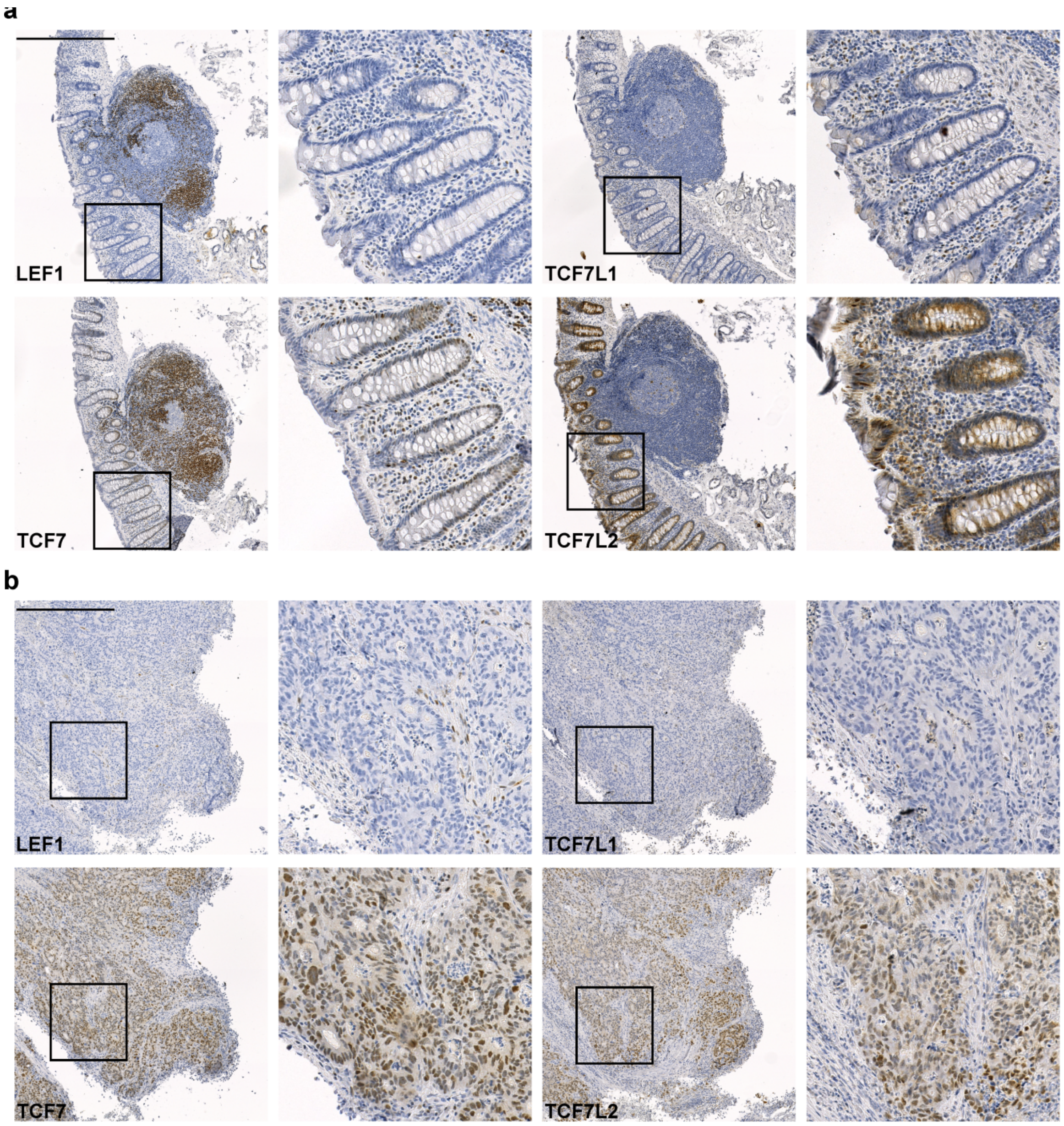

c

	fraction of samples staining positively for:			
	LEF1	TCF7	TCF7L1	TCF7L2
healthy	0/6	5/5	0/6	9/9
tumor	1/6	4/5	0/6	9/9

Supplementary Fig. S3 Expression of TCF/LEF family members in healthy colon epithelium and matched CRC tissue. **a,b** Formaldehyde-fixed and paraffin-embedded serial sections from human healthy colon tissue (a) and a case-matched colorectal tumor sample (b) were stained by immunohistochemistry with antibodies against LEF1, TCF7L1, TCF7, and TCF7L2. Counterstaining was performed using Mayer's hemalum solution. Representative pictures from one of at least five similarly processed pairs of tissue specimens are shown. The scale bar represents 500 µm. Boxes mark areas that are shown at 10-fold higher magnification on the right-hand side of the corresponding panel. **c** The table summarizes the number of samples that showed positive staining for the respective TCF/LEF proteins.

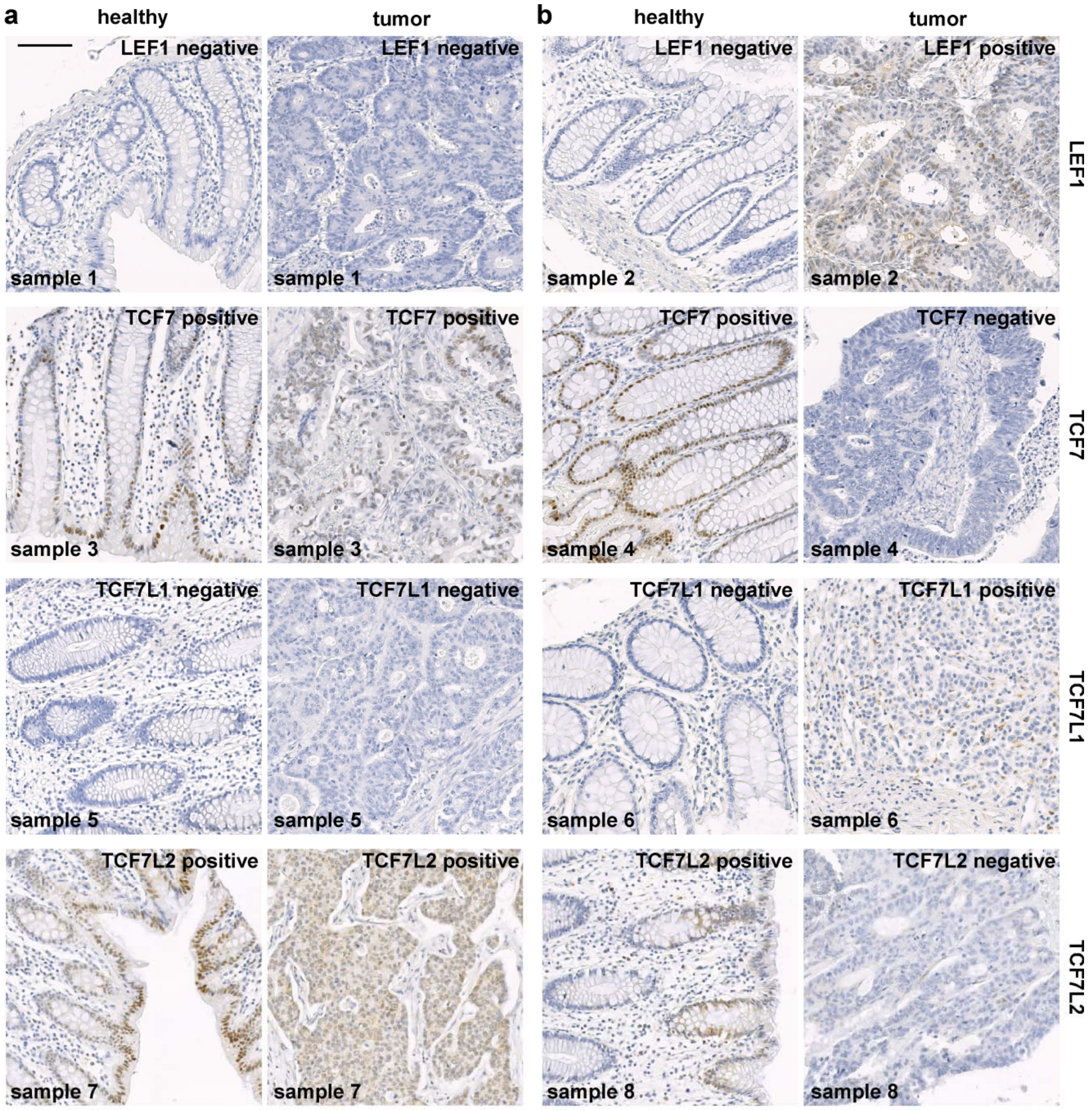

c

	fraction of samples staining positively for:			
	LEF1	TCF7	TCF7L1	TCF7L2
healthy	0/90	83/90	0*/90	88/90
tumor	8/90	34/90	6/90	70/90

*: one sample showed sporadic epithelial cells positive for TCF7L1

Supplementary Fig. S4 Expression of TCF/LEF family members in healthy colon epithelium and matched CRC tissue in a larger sample set of different origin. **a,b** A tissue microarray comprising sections of human adenocarcinoma tissue specimens with matched cancer-adjacent tissue or adjacent normal tissue was analyzed by immunohistochemistry with antibodies against LEF1, TCF7L1, TCF7, and TCF7L2. Counterstaining was performed using Mayer's hemalum solution. Micrographs show representative examples of cases with the same (a) and altered (b) expression of TCF/LEF family members in matching pairs of healthy and CRC tissue. The scale bar represents 100 µm. **c** The table summarizes the number of samples that showed positive staining for the respective TCF/LEF proteins.

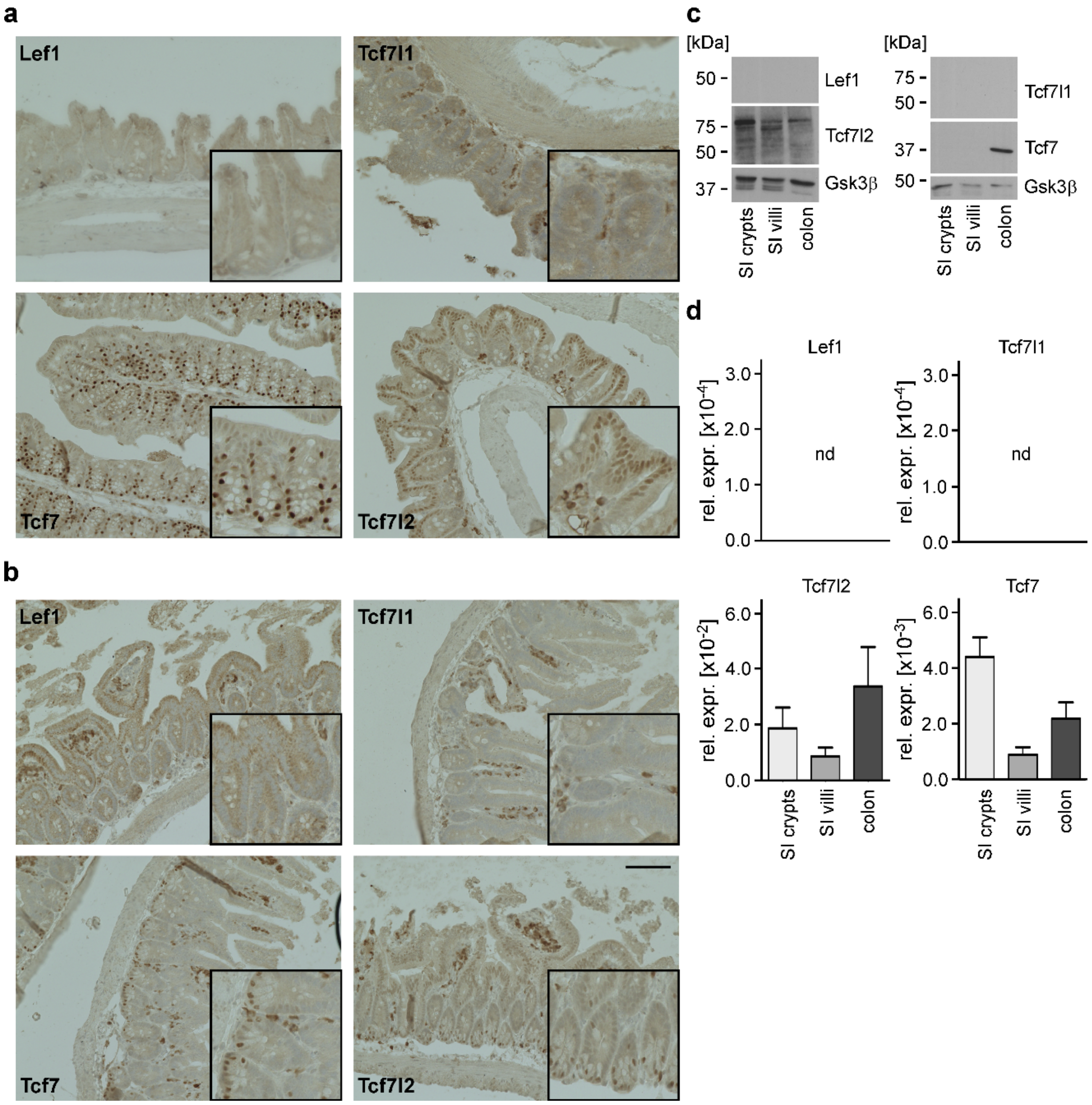

Supplementary Fig. S5 Tcf7l2 and Tcf7 are the predominant Tcf/Lef family members expressed in the healthy mouse intestinal epithelium. **a,b** The colon (a) and small intestine (b) of healthy C57BL/6N mice was fixed, sectioned and stained by immunohistochemistry with antibodies against Lef1, Tcf7l1, Tcf7, and Tcf7l2. Counterstaining was performed using Mayer's hemalum solution. Representative pictures from one of three independent biological replicates are shown. Scale bar represents 100 µm. Boxed inserts show selected areas at 4-fold higher magnification. **c** Western blot analyses to detect Tcf/Lef expression in nuclear extracts from epithelial cells of the small intestine (SI) and colon of healthy C57BL/6N mice. The small intestinal epithelium was fractionated into villus and crypt cells. GSK3β served as loading control. Molecular weights are given in kDa. Representative results from one of three independent biological replicates are shown. **d** RNA was isolated from epithelial cells of the small intestine (SI) and colon of healthy C57BL/6N mice, and qRT-PCR was performed to measure Tcf/Lef transcript levels. Data represent relative expression (rel. expr.) normalized to *Gapdh* transcripts. The mean values from at least three independent biological replicates and the corresponding SEM were plotted. nd: not detectable.

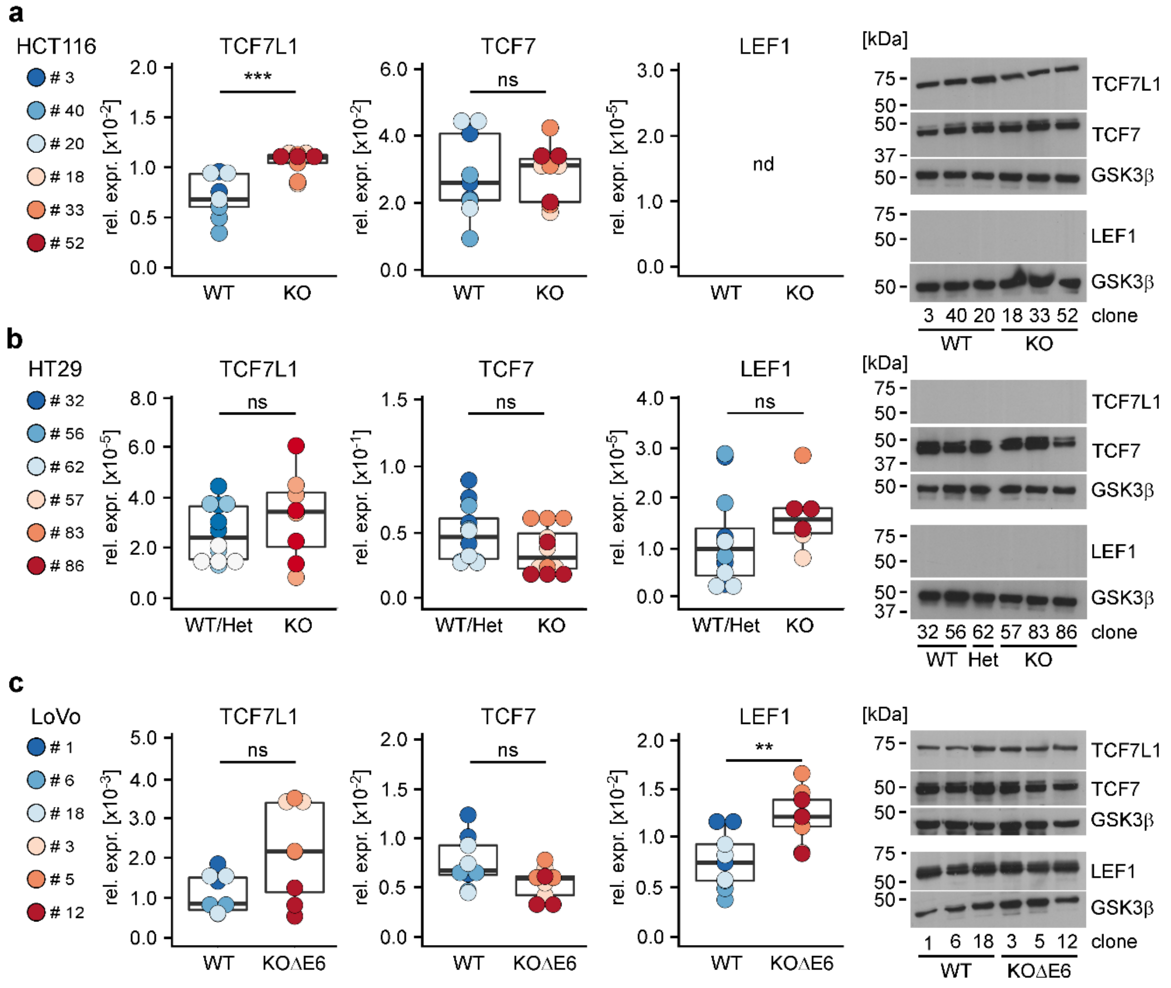

Supplementary Fig. S6 TCF7L2 deficiency does not lead to compensatory upregulation of *TCF7L1*, *TCF7*, and *LEF1*. **a-c** To investigate whether absence of *TCF7L2* leads to changes in the expression of *TCF7L1*, *TCF7*, and *LEF1*, qRT-PCR analyses and Western blotting with nuclear extracts were performed with HCT116 (a), HT29 (b) and LoVo cells (c). The status of the *TCF7L2* gene in the cell clones analyzed was biallelic WT, heterozygous (Het), and biallelic knock-out (KO) with intra-exon 6 mutations or exon 6 deletion (KOΔE6). For qRT-PCR, TCF/LEF expression was normalized to *GAPDH*, and relative expression levels (rel. expr.) are shown. Each dot represents an individual measurement, color of the dots identifies different cell clones. The box plots summarize expression data from *TCF7L2* WT/Het and KO cells. LMM analysis was performed for statistical analysis (n=3). For Western blot analyses, GSK3β served as loading control. Molecular weight is given in kDa. Representative results from one of three independent biological replicates are shown. nd: not detectable.

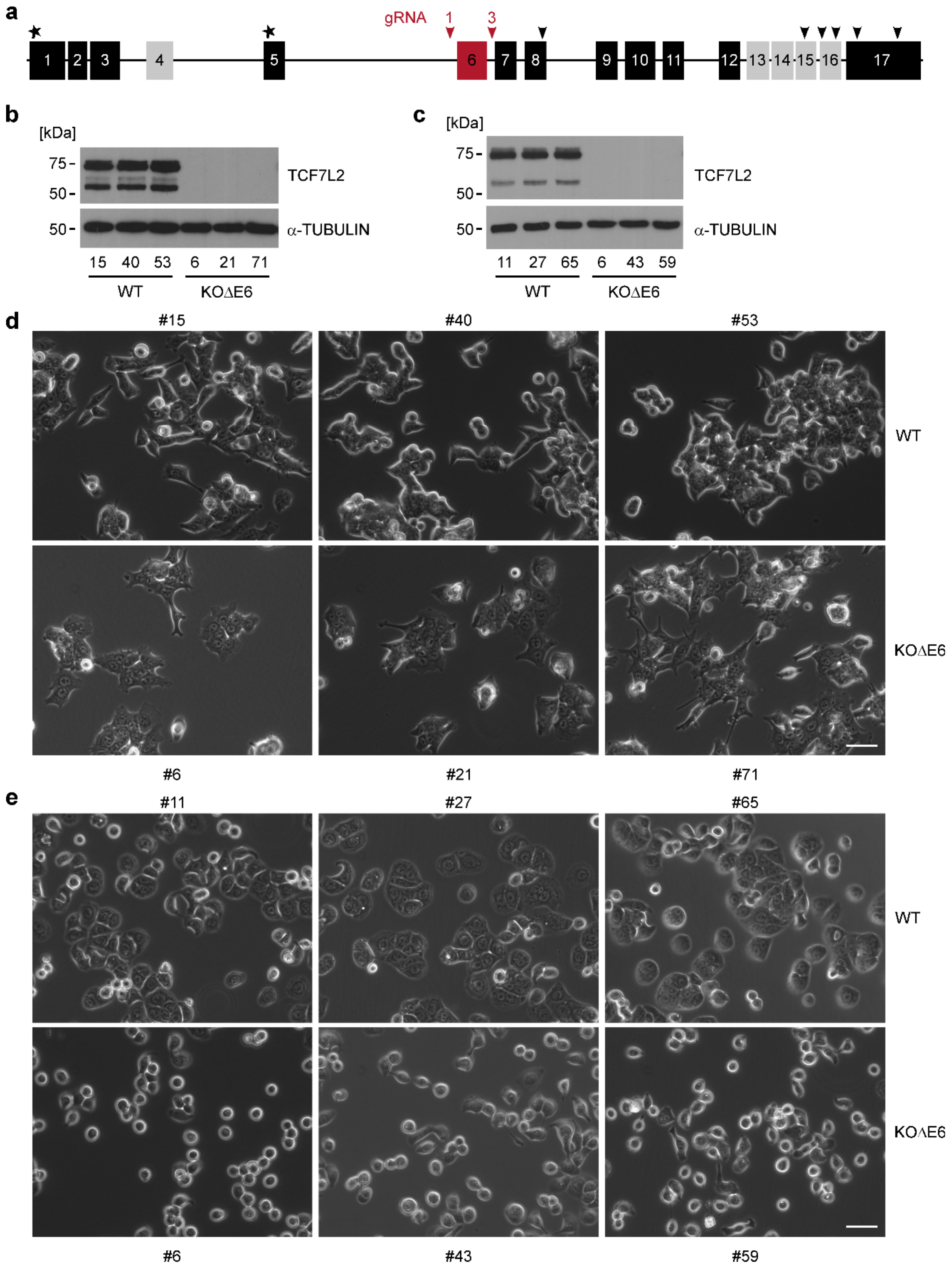

Supplementary Fig. S7 *TCF7L2* exon 6 deletion in HCT116 and HT29 cells results in morphological changes highly similar to intra-exon 6 mutations. **a** Scheme of the *TCF7L2* gene with its 17 exons (numbered boxes). Constitutively expressed exons are colored in black and red, alternatively spliced exons in grey. Asterisks mark start codons. Black arrowheads denote stop codons whose usage depends on the exon composition of alternatively spliced transcripts. The locations of gRNAs used to delete exon 6 are indicated by red arrowheads. **b,c** Western blot analysis to detect TCF7L2 expression in $TCF7L2^{WT}$ and $TCF7L2^{KO\Delta E6}$ clones in HCT116 (b) and HT29 (c). ΔE6: deletion of exon 6. α-TUBULIN was used as loading control. Molecular weights are given in kDa. Representative results from one of three independent biological replicates are shown. **d,e** Representative micrographs from one of three independent biological replicates showing the indicated $TCF7L2^{WT}$ and $TCF7L2^{KO\Delta E6}$

cell clones derived from HCT116 (d) and HT29 (e) cells 24 h after seeding the same starting numbers of cells. The scale bars represent 50 µm.

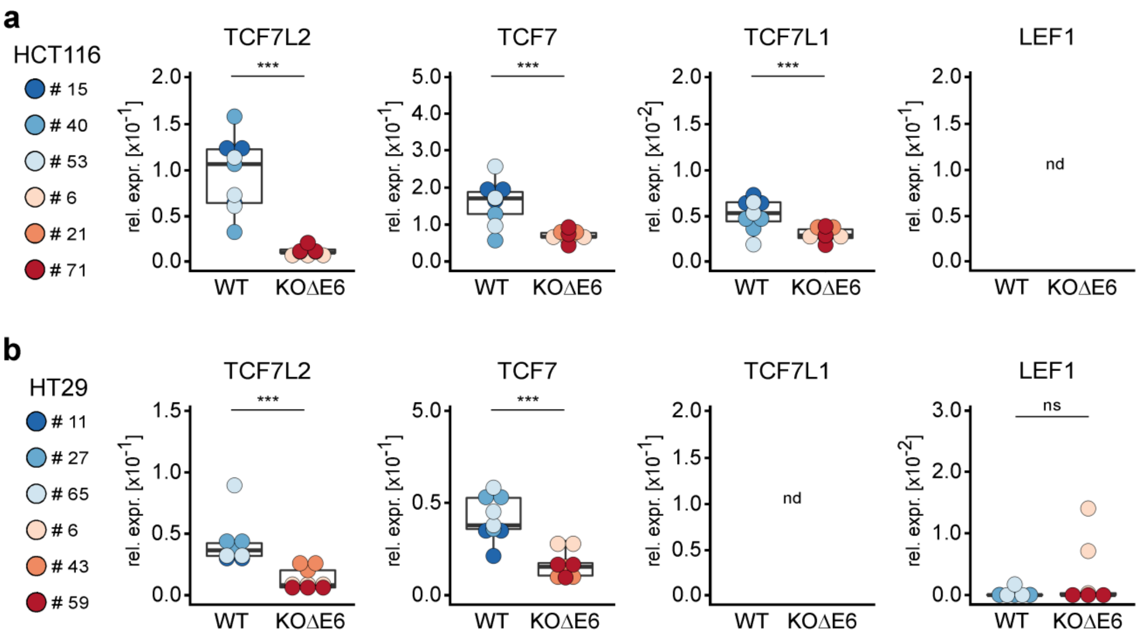

Supplementary Fig. S8 An alternative *TCF7L2* knockout strategy does not lead to increased expression of *TCF7L1*, *TCF7*, and *LEF1* in HCT116 and HT29 cells. **a,b** Expression of the genes indicated was analyzed in the newly generated HCT116 (a) and HT29 (b) $TCF7L2^{WT}$ and $TCF7L2^{KO\Delta E6}$ cells by qRT-PCR. Colored dots represent qRT-PCR results for individual cell clones. Box plots summarize qRT-PCR results from all clones according to *TCF7L2* genotype. Data presented indicate *TCF7L2* expression relative to that of *GAPDH* (rel. expr.). Linear mixed model (LMM) analysis was performed to assess statistical significance (n=3). ns: not significant; nd: not detectable.

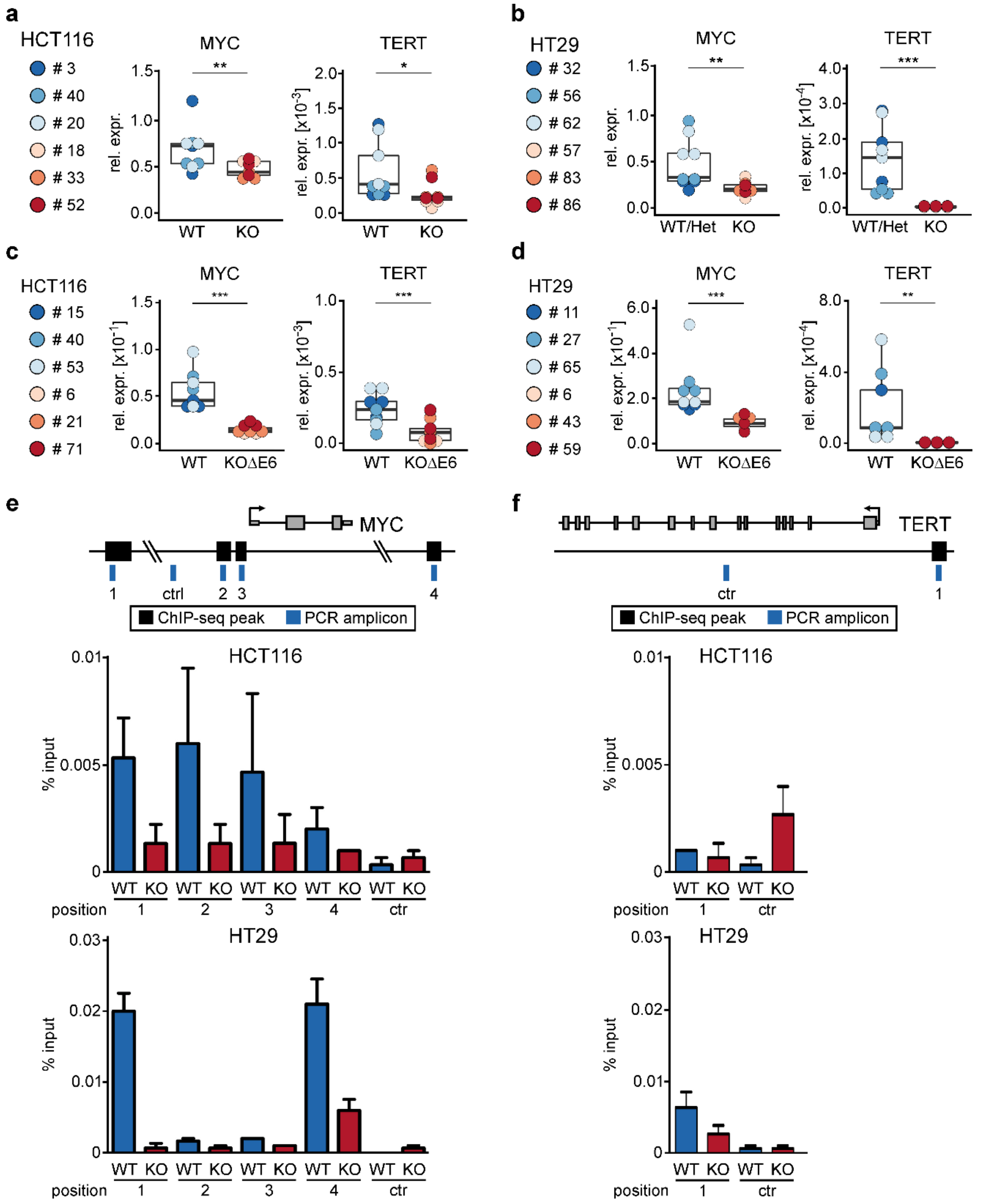

Supplementary Fig. S9 The Wnt/β-Catenin target genes *MYC* and *TERT* are downregulated in the absence of TCF7L2. **a-d** To investigate the effect of TCF7L2 on target genes of the Wnt/β-Catenin signaling pathway, the expression of *MYC* and *TERT* was analyzed in HCT116 (a,c) and HT29 (b,d) cell derivatives. The status of the *TCF7L2* gene in the cell clones analyzed was biallelic WT, heterozygous (Het), and biallelic knockout (KO) with intra-exon 6 mutations ($TCF7L2^{KO}$ [a,b]) or exon 6 deletion ($TCF7L2^{KO\Delta E6}$ [c,d]). For qRT-PCR, the expression was normalized to *GAPDH* transcripts and relative expression levels (rel. expr.) are shown. Each dot represents an individual measurement. Color of the dots identifies different cell clones. The box plots are used to summarize expression data from *TCF7L2* WT/Het and KO cells. LMM analysis was performed for statistical analysis (n=3). **e,f** To analyze potential binding of TCF7L2 to *MYC* and *TERT*, ChIP was performed. Gene models for *MYC* and *TERT* are depicted in the upper parts of the panels. Untranslated regions and exons are represented by gray boxes of different heights. The transcription start sites and the direction of transcription are indicated by angled arrows. Below the gene models, TCF7L2 ChIP-seq peak regions

and the corresponding amplicons analyzed by qPCR are shown. One WT (HCT116 # 3 and HT29 # 56) and one KO clone (HCT116 # 18 and HT29 # 57) from HCT116 and HT29 cells was analyzed. The results for *MYC* (e) and *TERT* (f) are depicted as bar plots and the values are shown as % input. The mean values from three independent biological replicates and the SEM were plotted.

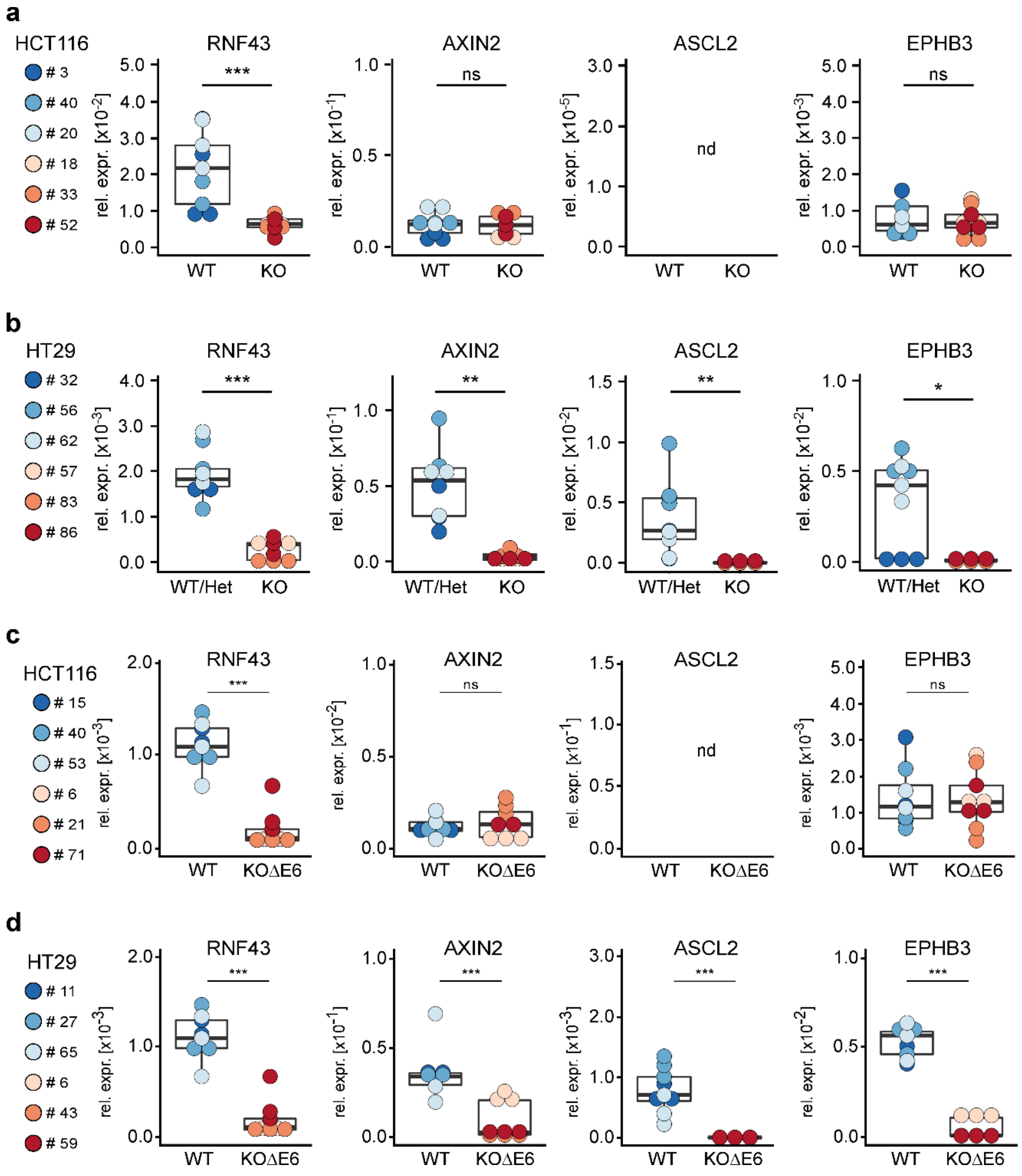

Supplementary Fig. S10 Inactivation of *TCF7L2* impairs WNT/β-Catenin target gene expression. **a,b** Expression of the *RNF43*, *AXIN2*, *ASCL2* and *EPHB3* genes was analyzed in HCT116 (a) and HT29 cells (b) with WT and mutant *TCF7L2*. For qRT-PCR, the expression was normalized to *GAPDH* transcripts and relative expression levels (rel. expr.) are shown. **c,d** WNT/β-Catenin target gene expression was analyzed by qRT-PCR in *TCF7L2* WT and mutant cells generated by an alternative knockout strategy (KOΔE6). Gene expression was normalized to *GAPDH* transcripts and relative expression levels (rel. expr.) are shown. **a-d** Each dot represents an individual measurement. Color of the dots identifies different cell clones. The box plots are used to summarize expression data from *TCF7L2* WT/Het and mutant cells. nd: not detectable. LMM analysis was performed to assess statistical significance; n=3 for all experiments shown.

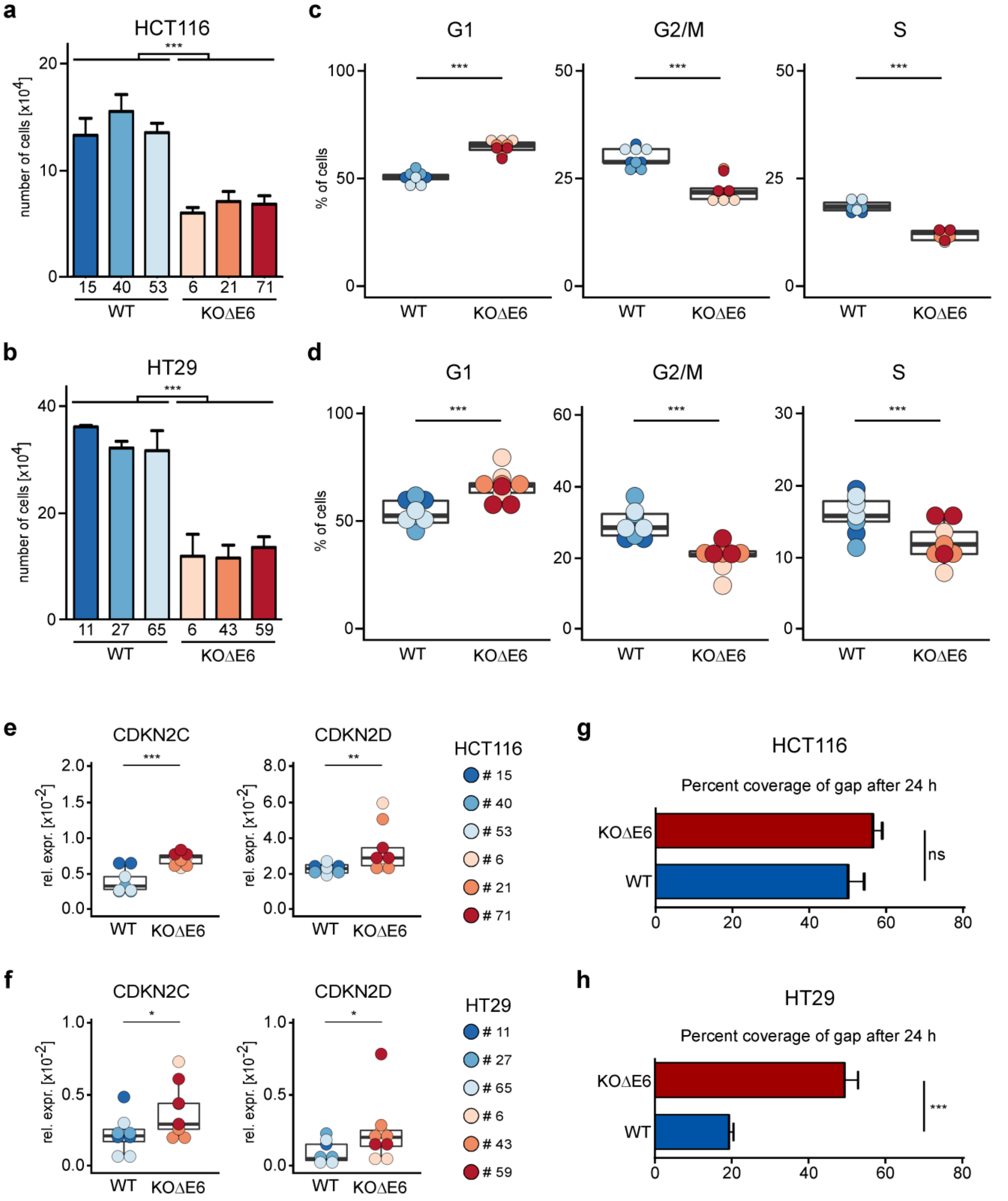

Supplementary Fig. S11 Changes in proliferation, cell cycle and migration are independent of the gRNAs used for inactivation of *TCF7L2* in HCT116 and HT29 cells. **a,b** $1x10^4$ HCT116 (a) and $5x10^4$ HT29 (b) cells with the genotypes indicated were seeded, and incubated for 72 h. Thereafter, the resulting cell counts were determined and displayed as bar plots. **c,d** To analyze differences in cell cycle distribution of $TCF7L2^{WT}$ and $TCF7L2^{KO\Delta E6}$ cells in HCT116 (c) and HT29 (d), cells were stained with propidium iodide and analyzed by flow cytometry. The proportions of cells in different cell cycle phases are depicted by the box plots. **e,f** Expression of the cell cycle inhibitors *CDKN2C* and *CDKN2D* was analyzed in HCT116 (e) and HT29 cells (f) by qRT-PC. Colored dots represent qRT-PCR results for individual cell clones. Box plots summarize qRT-PCR results from all clones according to *TCF7L2* genotype. Data presented indicate *TCF7L2* expression relative to that of *GAPDH* (rel. expr.). **g,h** The migratory capacity of HCT116 (g) and HT29 (h) $TCF7L2^{WT}$ and $TCF7L2^{KO\Delta E6}$ cells was examined by gap closure assays. Bar graphs depict the percent coverage of the gaps at 24 h after infliction. Shown are the combined mean values for all clones with the genotypes indicated. Error bars represent the SEM. **a-h** To assess statistical significance, LMM analysis was applied; n=3 for all experiments shown.

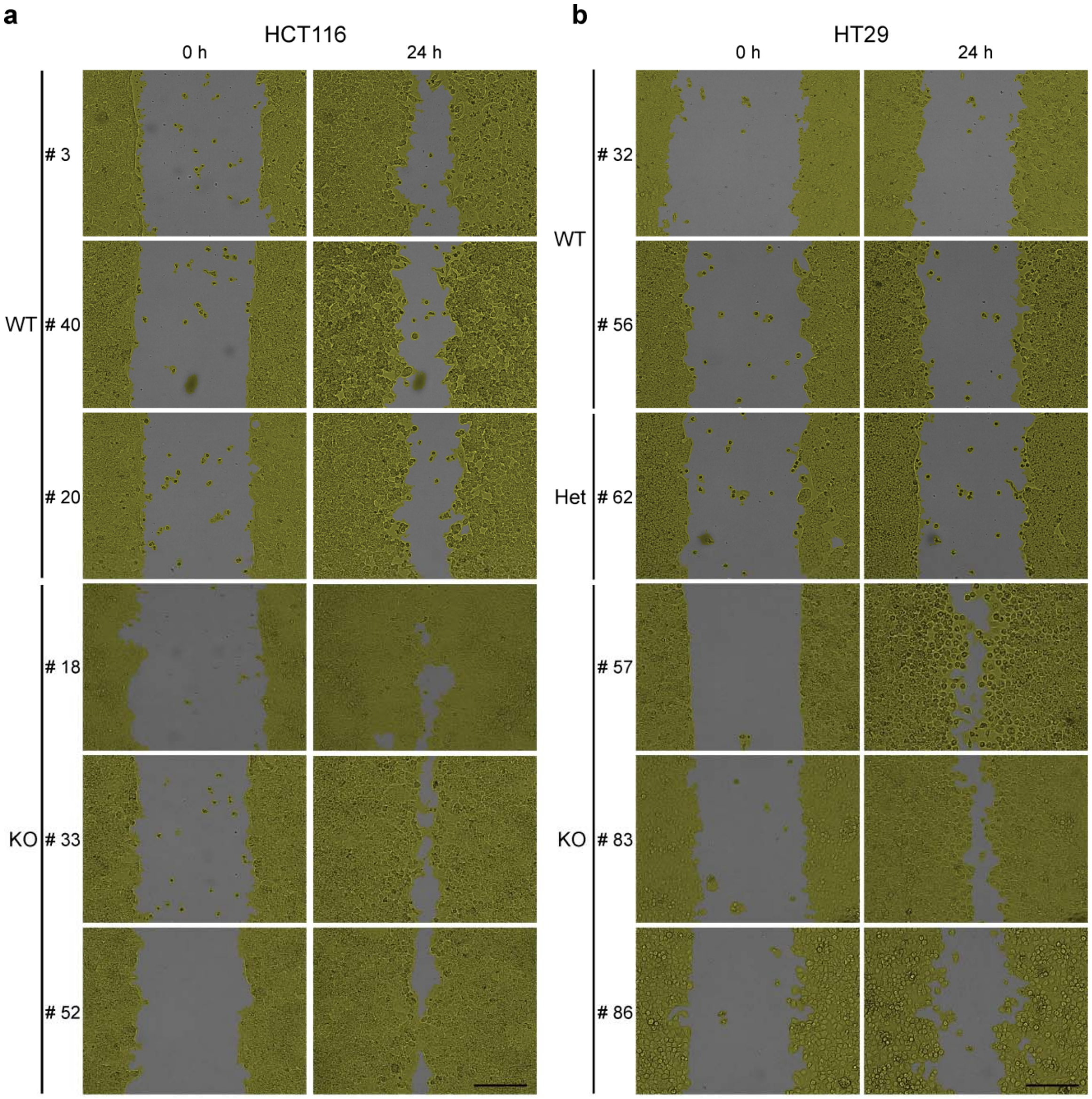

Supplementary Fig. S12 *TCF7L2* KO cells show enhanced migration. **a,b** HCT116 (a) and HT29 (b) cells with WT and mutant *TCF7L2* were seeded into the chambers of ibidi® cell culture inserts. After overnight incubation, the cells were treated with mitomycin C for 45 min before the ibidi® cell culture inserts were removed, thereby creating a cell-free area of 100 µm width. For every cell clone, four independent analyses were performed. Representative images from one of the four independent biological replicates show cell-free areas immediately after removal of ibidi® cell culture inserts (0 h) and 24 h thereafter. The scale bars represent 100 µm.

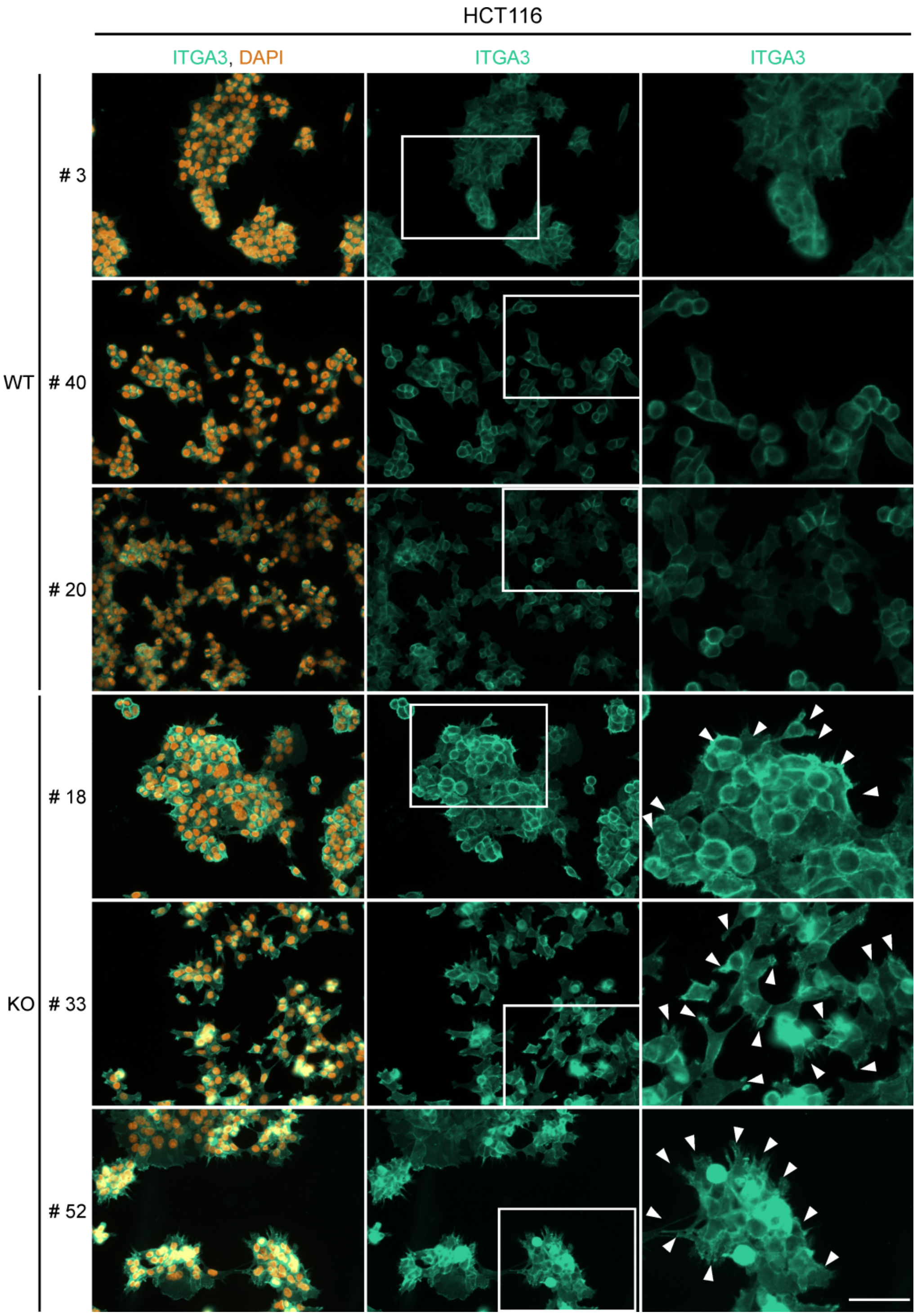

Supplementary Fig. S13 ITGA3 is upregulated and marks cell protrusions in *TCF7L2*-deficient HCT116 cells. HCT116 cell clones with WT and mutant *TCF7L2* were seeded on gelatin-coated glass slides and incubated for 24 h. Then, the cells were fixed and stained with antibodies directed against ITGA3. DAPI was used to stain nuclei (orange). For each cell clone, representative images from one of three independent biological replicates are shown. The scale bar represents 50 µm. Panels on the right show higher magnifications of areas marked with white frames in pictures in the center column. White arrowheads indicate ITGA3-positive cell protrusions.

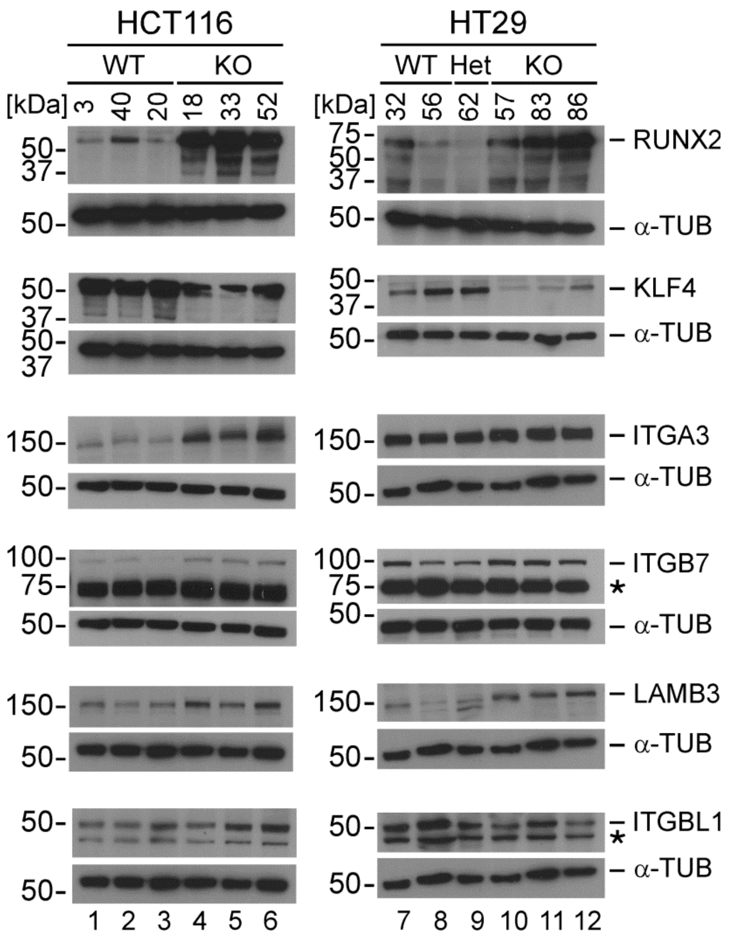

Supplementary Fig. S14 Loss of TCF7L2 leads to changes in expression of transcription factors and cell-adhesion molecules. Western blot analyses using whole cell lysates were performed in HCT116 and HT29 TCF7L2 WT/Het and KO cells to detect RUNX2, KLF4, ITGA3, ITGB7, LAMB3, and ITGBL1 expression. α-TUBULIN (α-TUB) served as loading control. For HCT116 cells, ITGA3, ITGB7, LAMB3, and ITGBL1 were sequentially detected on the same blot. For HT29 cells, ITGA3, LAMB3, and ITGBL1 were sequentially analyzed on the same blot. Asterisks mark unspecific bands. Molecular weight is given in kDa. Representative pictures from one of three independent biological replicates are shown.

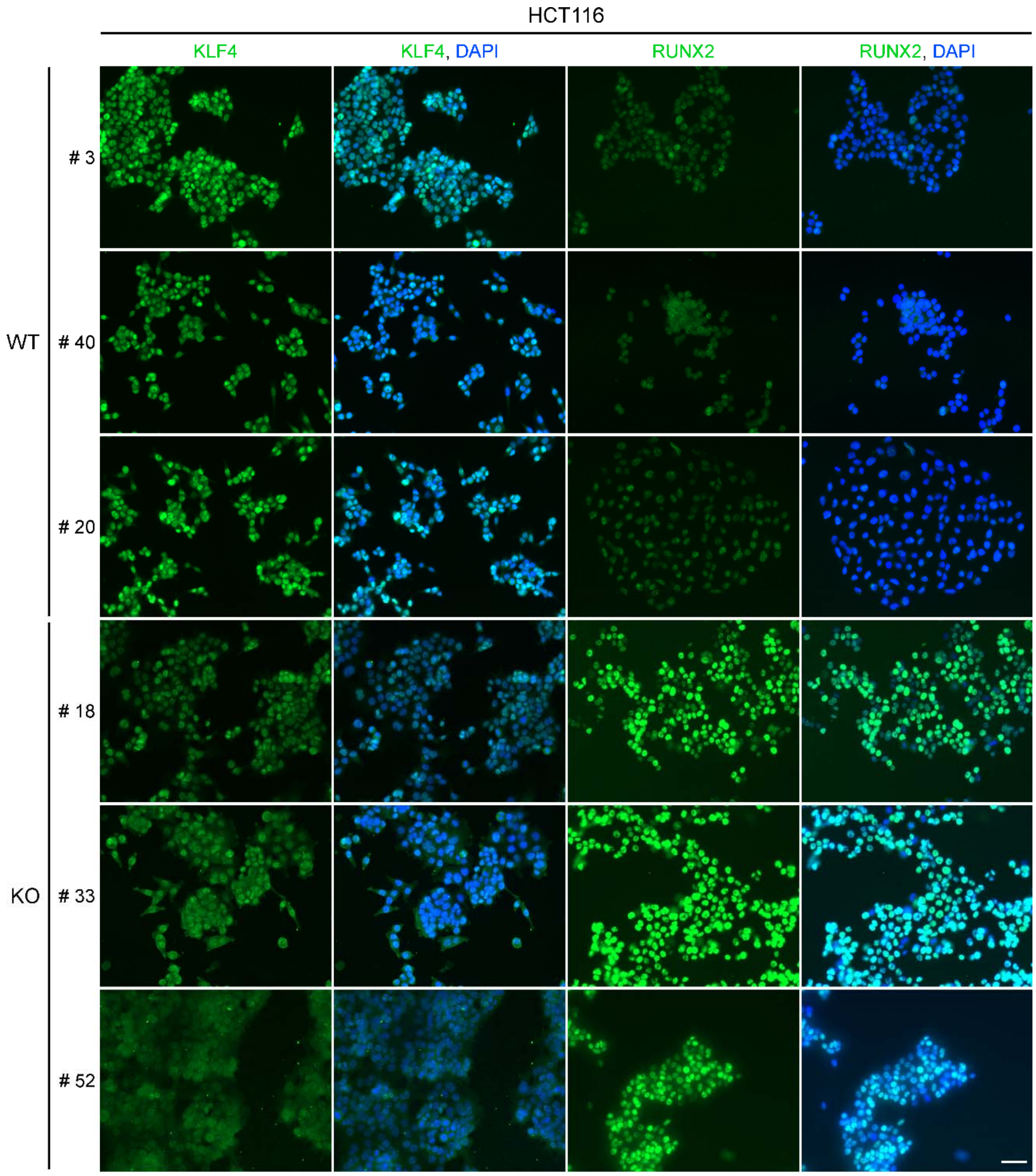

Supplementary Fig. S15 Up- and downregulation of RUNX2 and KLF4, respectively, in HCT116 cells upon knock-out of *TCF7L2*. HCT116 cell clones with WT and mutant *TCF7L2* were seeded on gelatin-coated glass slides and incubated for 24 h. Then, the cells were fixed and stained with antibodies directed against KLF4 (left panels) and RUNX2 (right panels). DAPI was used to stain nuclei (blue color). For each cell clone, representative images from one of three independent biological replicates are shown. Scale bar represents 50 µm.

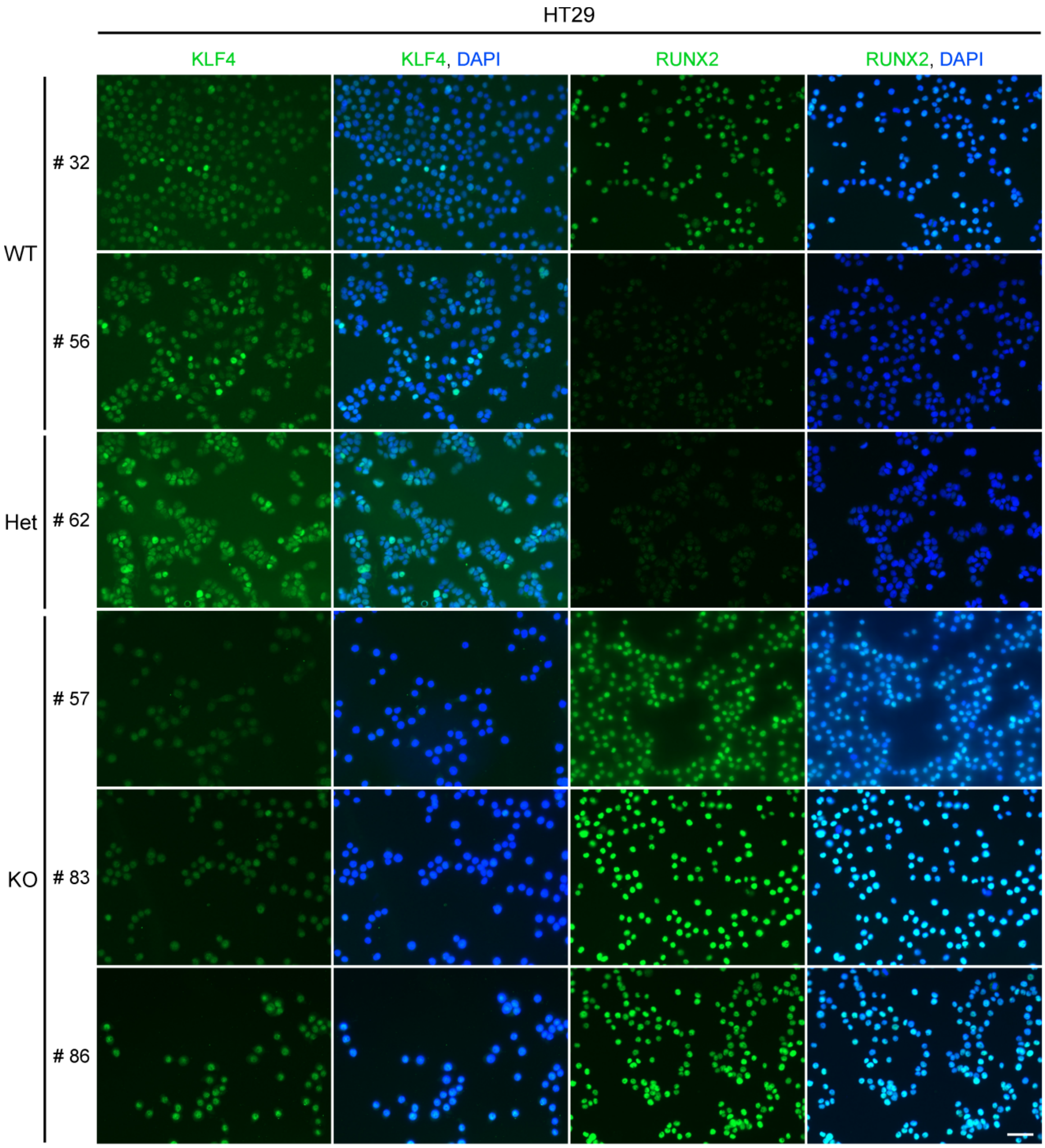

Supplementary Fig. S16 Up- and downregulation of RUNX2 and KLF4, respectively, in HT29 cells upon knock-out of *TCF7L2*. HT29 cell clones with WT and mutant *TCF7L2* were seeded on gelatin-coated glass slides and incubated for 24 h. Then, the cells were fixed and stained with antibodies directed against KLF4 (left panels) and RUNX2 (right panels). DAPI was used to stain nuclei (blue color). For each cell clone, representative images from one of three independent biological replicates are shown. Scale bar represents 50 µm.

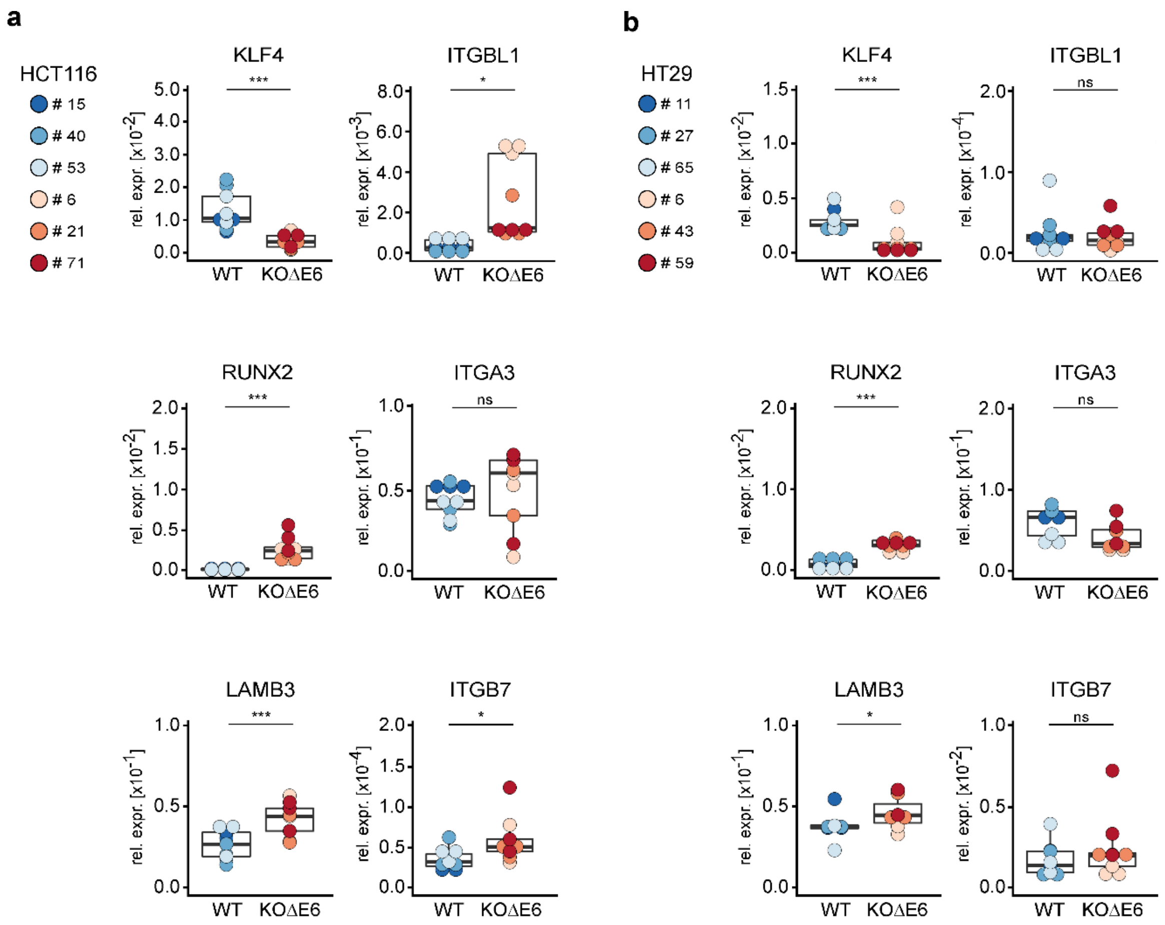

Supplementary Fig. S17 Gene expression changes of *KLF4*, *RUNX2* and cell adhesion factors upon inactivation of *TCF7L2* in HCT116 and HT29 cells. Expression of the genes indicated was analyzed in HCT116 (**a**) and HT29 (**b**) *TCF7L2*^{WT} and *TCF7L2*$^{KO\Delta E6}$ cells by qRT-PCR. Colored dots represent qRT-PCR results for individual cell clones. Box plots summarize qRT-PCR results from all clones according to *TCF7L2* genotype. Data presented indicate *TCF7L2* expression relative to that of *GAPDH* (rel. expr.). Linear mixed model (LMM) analysis was performed to assess statistical significance (n=3). ns: not significant.

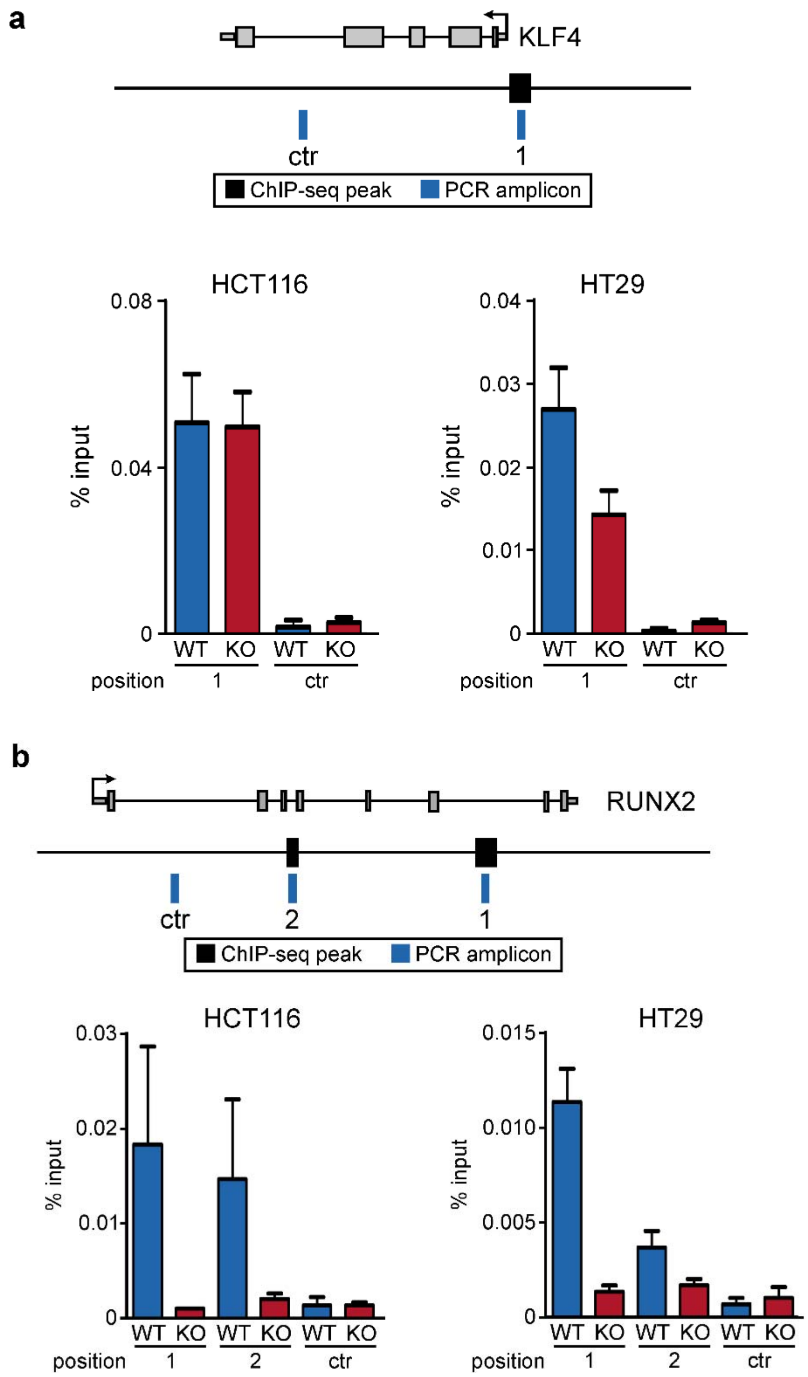

Supplementary Fig. S18 The transcription factor *RUNX2* is a direct target gene of TCF7L2. **a,b** To investigate binding of TCF7L2 to *KLF4* and *RUNX2*, ChIP was performed. Gene models for *KLF4* and *RUNX2* are depicted in the upper parts of the panels. Untranslated regions and exons are represented by gray boxes of different heights. The transcription start sites and the direction of transcription are indicated by angled arrows. Below the gene models, TCF7L2 ChIP-seq peak regions and the corresponding amplicons analyzed by qPCR are shown. One WT (HCT116 # 3 and HT29 # 56) and one KO clone (HCT116 # 18 and HT29 # 57) from HCT116 and HT29 cells was analyzed. The results for *KLF4* (a) and *RUNX2* (b) are depicted as bar plots and the values are shown as % input. The mean values from three independent biological replicates and the SEM were plotted.
