# Supplementary material for: Loss of the nuclear Wnt pathway effector TCF7L2 promotes migration and invasion of human colorectal cancer cells
Source: Oncogene. 2020 Mar 20;39(19):3893–909. doi: 10.1038/s41388-020-1259-7 (PMC7203011; doi:10.1038/s41388-020-1259-7)
Supplement: Supplementary file 2 — Supplementary table S1 [file 41388_2020_1259_MOESM2_ESM.docx]

**Supplementary table S1: Mutations in CRC driver pathway genes, MSI/MSS status, and CMS classification of cell lines used in this study**

| **Gene / feature** | **Cell line** | | | | |
| --- | --- | --- | --- | --- | --- |
|  | **HCT116** | **HT29** | **LoVo** | **LS174T** | **SW480** |
| *APC* | WT | MUT | MUT | WT | MUT |
| *CTNNB1* | MUT | WT | WT | MUT | WT |
| *KRAS* | MUT | WT | MUT | MUT | MUT |
| *BRAF* | WT | MUT | WT | WT | WT |
| *PIK3CA* | MUT | MUT | WT | MUT | WT |
| *SMAD4* | WT | MUT | WT | WT | MUT |
| *TGFBR2* | MUT | WT | MUT | MUT | WT |
| *TP53* | WT | MUT | WT | WT | MUT |
| MSI/MSS status | MSI | MSS | MSI | MSI | MSS |
| CMS* | 4 | 3 | 1 | 3 | 4 |

*CMS: consensus molecular subtype
