# Supplementary material for: Loss of the nuclear Wnt pathway effector TCF7L2 promotes migration and invasion of human colorectal cancer cells
Source: Oncogene. 2020 Mar 20;39(19):3893–909. doi: 10.1038/s41388-020-1259-7 (PMC7203011; doi:10.1038/s41388-020-1259-7)
Supplement: Supplementary file 3 — Supplementary table S2 [file 41388_2020_1259_MOESM3_ESM.docx]

**Supplementary table S2: Summary of targeting strategy and genomic status of *TCF7L2* exon 6 (ENSE00003789188)**

| **Cell line** | **Clone** | **gRNA target(s)** | ***TCF7L2* status** | **Allele 1** | **Allele 2** |
| --- | --- | --- | --- | --- | --- |
| HCT116 | # 3 | exon 6 | WT^a^ | WT | WT |
| HCT116 | # 40 | exon 6 | WT^a^ | WT | WT |
| HCT116 | # 20 | exon 6 | WT^a^ | WT | WT |
| HCT116 | # 18 | exon 6 | KO^a^ | 1 bp insertion at position 114^b^ | 149 bp deletion at position 114^b^ |
| HCT116 | # 33 | exon 6 | KO^a^ | 160 bp insertion at position 114^b^ | 16 bp deletion at position 114^b^ |
| HCT116 | # 52 | exon 6 | KO^a^ | 32 bp deletion at position 90^b^ | 38 bp deletion at position 86^b^ |
| HCT116 | # 15 | intron 5 + intron 6 | WT^a^ | WT | WT |
| HCT116 | # 40 | intron 5 + intron 6 | WT^a^ | WT | WT |
| HCT116 | # 53 | intron 5 + intron 6 | WT^a^ | WT | WT |
| HCT116 | # 6 | intron 5 + intron 6 | KO | Δ ENSE00003789188 | Δ ENSE00003789188 |
| HCT116 | # 21 | intron 5 + intron 6 | KO | Δ ENSE00003789188 | Δ ENSE00003789188 |
| HCT116 | # 71 | intron 5 + intron 6 | KO | Δ ENSE00003789188 | Δ ENSE00003789188 |
| HT29 | # 32 | exon 6 | WT^a^ | WT | WT |
| HT29 | # 56 | exon 6 | WT^a^ | WT | WT |
| HT29 | # 62 | exon 6 | Het^a^ | WT | 1 bp deletion at position 114^b^ |
| HT29 | # 57 | exon 6 | KO^a^ | 1 bp deletion at position 114^b^ | 10 bp deletion at position 114^b^ |
| HT29 | # 83 | exon 6 | KO^a^ | 10 bp deletion at position 114^b^ | 1 bp deletion at position 114^b^ |
| HT29 | # 86 | exon 6 | KO^a^ | 26 bp deletion at position 98^b^ | 26 bp deletion at position 98^b^ |
| HT29 | # 11 | intron 5 + intron 6 | WT^a^ | WT | WT |
| HT29 | # 27 | intron 5 + intron 6 | WT^a^ | WT | WT |
| HT29 | # 65 | intron 5 + intron 6 | WT^a^ | WT | WT |
| HT29 | # 6 | intron 5 + intron 6 | KO^a^ | Δ ENSE00003789188 | Δ ENSE00003789188 |
| HT29 | # 43 | intron 5 + intron 6 | KO^a^ | Δ ENSE00003789188 | Δ ENSE00003789188 |
| HT29 | # 59 | intron 5 + intron 6 | KO | Δ ENSE00003789188 | Δ ENSE00003789188 |
| LoVo | # 1 | intron 5 + intron 6 | WT^a^ | WT | WT |
| LoVo | # 6 | intron 5 + intron 6 | WT^a^ | WT | WT |
| LoVo | # 18 | intron 5 + intron 6 | WT^a^ | WT | WT |
| LoVo | # 3 | intron 5 + intron 6 | KO | Δ ENSE00003789188 | Δ ENSE00003789188 |
| LoVo | # 5 | intron 5 + intron 6 | KO | Δ ENSE00003789188 | Δ ENSE00003789188 |
| LoVo | # 12 | intron 5 + intron 6 | KO | Δ ENSE00003789188 | Δ ENSE00003789188 |

^a^ determined by sequencing

^b^ coordinates refer to the 5´-end of ENSE00003789188
