# Supplementary material for: Loss of the nuclear Wnt pathway effector TCF7L2 promotes migration and invasion of human colorectal cancer cells
Source: Oncogene. 2020 Mar 20;39(19):3893–909. doi: 10.1038/s41388-020-1259-7 (PMC7203011; doi:10.1038/s41388-020-1259-7)
Supplement: Supplementary file 8 — Supplementary table S7 [file 41388_2020_1259_MOESM8_ESM.docx]

**Supplementary table S7: Number of colon adenocarcinoma patients per TNM and AJCC staging category***

| T | | | | | N | | M | | AJCC stage | | | |
| --- | --- | --- | --- | --- | --- | --- | --- | --- | --- | --- | --- | --- |
| Tis | 1 | 2 | 3 | 4 | 0 | >0 | 0 | 1 | I | II | III | IV |
| 1 | 10 | 55 | 244 | 41 | 206 | 145 | 249 | 48 | 55 | 135 | 102 | 48 |

* A total number of 351 colon adenocarcinoma patients from the TCGA COAD cohort was assigned to TNM and AJCC staging categories depending upon availability of clinical data.
