# Supplementary material for: Loss of the nuclear Wnt pathway effector TCF7L2 promotes migration and invasion of human colorectal cancer cells
Source: Oncogene. 2020 Mar 20;39(19):3893–909. doi: 10.1038/s41388-020-1259-7 (PMC7203011; doi:10.1038/s41388-020-1259-7)
Supplement: Supplementary file 9 — Supplementary table S8 [file 41388_2020_1259_MOESM9_ESM.docx]

**Supplementary table S8: Cell lines used in this study*^,#^**

| **cell line** | **source** | **culture conditions** |
| --- | --- | --- |
| HCT116 | Max-Planck-Institute for Immunology and Epigenetics (Freiburg, Germany) | DMEM supplemented with 10% (v/v) FCS, 10 mM HEPES, 1% (v/v) MEM non-essential amino acids solution, and 1% (v/v) penicillin/streptomycin at 37°C and 5% CO_2_. |
| HT29 | German Cancer Research Center Cell Line Service (Heidelberg, Germany) |  |
| LoVo | CLS Cell Lines Service GmbH (Eppelheim, Germany) |  |
| LS174T | German Cancer Research Center Cell Line Service (Heidelberg, Germany) |  |
| SW480 | Max-Planck-Institute for Immunology and Epigenetics (Freiburg, Germany) |  |

* Cell line identity was determined by SNP-profiling at Multiplexion Inc. (Friedrichshafen, Germany)

**^#^** Cell lines were routinely tested for mycoplasm contamination using the Myco sensor PCR assay kit

from Stratagene (San Diego, CA, USA)
