# Supplementary material for: Loss of the nuclear Wnt pathway effector TCF7L2 promotes migration and invasion of human colorectal cancer cells
Source: Oncogene. 2020 Mar 20;39(19):3893–909. doi: 10.1038/s41388-020-1259-7 (PMC7203011; doi:10.1038/s41388-020-1259-7)
Supplement: Supplementary file 10 — Supplementary table S9 [file 41388_2020_1259_MOESM10_ESM.docx]

**Supplementary table S9: List of Primers**

| **Gene** | **Forward primer (5´ - 3´)** | **Reverse primer (5´ - 3´)** |
| --- | --- | --- |
| ASCL2 | TGACCTGGGGCGTAATAAAG | ACACAGGCTTCTCCCTAGCA |
| AXIN2 | TGCTTTCGTGGAAATGACAG | AGGTGTGTGGAGGAAAGGTG |
| CDKN2C | TCATCCATGATGCGGCCAGA | GAAGATTTGTGGCTCCCCCA |
| CDKN2D | CCAATGTCCAGGACACCTCC | ACAGCAGTGTGACCCTCTTG |
| EPHB3 | GCTGGACTTTCGGACTCTTG | CACCCCCTCTCCTAATCCAT |
| GAPDH | ACCACAGTCCATGCCATCACT | GTCCACCACCCTGTTGCTGTA |
| ITGA3 | ACTGTGTGTGGCTAGAGTGC | CACCAGCTCCGAGTCAATGT |
| ITGB7 | AGAGGGAGGGTAAGGCTGAG | GCTCCTCTGAGAAGCCAAGG |
| ITGBL1 | CAAAGCAGGCTGGTATGGGA | CACAAACACAGCGACCACAG |
| KLF4 | GGGAAGGGAGAAGACACTGC | TCCAGGTCCAGGAGATCGTT |
| KLF4  (ChIP ctrl*) | CAAACCGGCGCCTACTTTTC | CGGGGAATCTCCTGCCTTTT |
| KLF4  (ChIP peak 1) | ACACGTTCGTTCTCTCTGGTC | GAGAGAAGAAAGGGAGGGGC |
| LEF1 (human) | ACAGATCACCCCACCTCTTG | TGAGGCTTCACGTGCATTAG |
| Lef1 (mouse) | TATGAACAGCGACCCGTACA | TCGTCGCTGTAGGTGATGAG |
| MYC | AAGAGGACTTGTTGCGGAAA | CTCAGCCAAGGTTGTGAGGT |
| MYC  (ChIP ctrl) | TTCTCTTCCTTCACCTCATCTC | AAAGCCAGCGAACTTAGGG |
| MYC  (ChIP peak 1) | CGTCCTTTGAGCTCAGCAGA | CCAGTCTAAGGCCCCAATCC |
| MYC  (ChIP peak 2) | CAAGGGTCTCTGCTGACTCC | GAGACAAATCCCCTTTGCGC |
| MYC  (ChIP peak 3) | CCACTCTCCCTGGGACTCTT | TGCTCCTCCGTAGCAGTACT |
| MYC  (ChIP peak 4) | TGACGGATGAGCACATGGTT | TCCCAAGCAGGAGCAGTTTC |
| RNF43 | CTGCTACCAGAAACCCCAGG | CTGCGGTGTCAGAACTCCAT |
| RUNX2 | CTTCCTGCCATCACCGATGT | TGCATTCGTGGGTTGGAGAA |
| RUNX2  (ChIP ctrl) | TGCTCAGAGCCAAGACATCA | TGGCACAGTGGGTTTTCAGT |
| RUNX2  (ChIP peak 1) | ATCTCGCGCTGTACCCAAAA | ATCACTGCTCTCCTTGGCAC |
| RUNX2  (ChIP peak 1) | CAAACAGAGCTGACCCCCTC | CCTCTGGCAGCAGACTGTAG |
| TCF7 (human) | AGCCAAGGTCATTGCAGAGT | GTGGTGGATTCTTGGTGCTT |
| Tcf7 (mouse) | GGAAAAAGAAATGCATTCGGTA | GTGAGTCCTGGGGAGCTG |
| TCF7L1 (human) | GGGTACCCCTTCCTGATGAT | GATGGTGACCTCGTGTCCTT |
| Tcf7l1 (mouse) | CGGGACAACTATGGGAAGAA | TTGCTCTTAGAGGCCAGAGC |
| TCF7L2 (human) | AGAAAAGAAGAAGCCCCACA | CGGGCCAGCTCGTAGTATT |
| Tcf7l2 (mouse) | TGCGTTCGCTACATACAAGG | TGGGTCTGCTCAGTCTGTGA |
| TERT | CTACGGCGACATGGAGAACA | AGAGATGACGCGCAGGAAAA |
| TERT  (ChIP ctrl) | ACTCGCAGGGGAATTCCAAG | TGGGCTCCCTGTTCTGTTTC |
| TERT  (ChIP peak1) | AAAGAAAGGAGAGGCAGGCG | CCAGCTGCTGGTGACTGTTA |

* ctrl: control region
