# Supplementary material for: Loss of the nuclear Wnt pathway effector TCF7L2 promotes migration and invasion of human colorectal cancer cells
Source: Oncogene. 2020 Mar 20;39(19):3893–909. doi: 10.1038/s41388-020-1259-7 (PMC7203011; doi:10.1038/s41388-020-1259-7)
Supplement: Supplementary file 11 — Supplementary table S10 [file 41388_2020_1259_MOESM11_ESM.docx]

**Supplementary table S10: List of Antibodies**

| **Antigen** | **Species of origin** | **Working dilution** | | | **Supplier**  **(catalogue no.)** |
| --- | --- | --- | --- | --- | --- |
|  |  | **WB*** | **IHC^§^, IF^#^** | **ChIP** |  |
| α-TUBULIN | mouse | 1:10 000 | - | - | Sigma-Aldrich (T9026) |
| CDKN2C | mouse | 1:1000 | - | - | Cell Signaling Technology (DCS118) |
| CDKN2D | mouse | 1:1000 | - | - | Invitrogen  (#378700) |
| GSK3β | mouse | 1:1000 | - | - | BD Biosciences (#610201) |
| ITGA3 | mouse | - | 1: 40 | - | Invitrogen  (# 17049442) |
| ITGA3 | mouse | 1:2500 | - | - | Proteintech Europe (66070-1-Ig) |
| ITGB7 | rabbit | 1:1000 | - | - | Proteintech Europe (11328-1-AP) |
| ITGBL1 | rabbit | 1:1000 | - | - | Thermo Fisher Life Technologies GmbH (#PA542123) |
| KLF4 | rabbit | 1:1000 | 1:200 | - | Cell Signaling Technology (D1F2) |
| LAMB3 | rabbit | 1:1000 | - | - | Proteintech Europe (26795-1-AP) |
| LEF1 | rabbit | 1:1000 | 1:100 | - | Cell Signaling Technology (C12A5) |
| RUNX2 | rabbit | 1:1000 | 1:200 | - | Cell Signaling Technology (D1L7F) |
| TCF7 | rabbit | 1:1000 | 1:200 | - | Cell Signaling Technology (C63D9) |
| TCF7L1 | rabbit | 1:1000 | 1:100 | - | Cell Signaling Technology (D15G11) |
| TCF7L2 | rabbit | 1:1000 | 1:100 | - | Cell Signaling Technology (C9B9) |
| TCF7L2 | rabbit | - | 1:100 | - | Cell Signaling Technology (C48H11) |
| TCF7L2 | rabbit | - | - | 1:50 | Thermo Fisher Life Technologies GmbH (#MA5-14935; lot UE2779552) |

*WB: Western blotting

^§^IHC: Immunohistochemistry

^#^IF: Immunofluorescence
